# A Reusable Efficient Green Catalyst of 2D Cu-MOF for the Click and Knoevenagel Reaction

**DOI:** 10.3390/molecules26175296

**Published:** 2021-08-31

**Authors:** Kaushik Naskar, Suvendu Maity, Himadri Sekhar Maity, Chittaranjan Sinha

**Affiliations:** 1 Department of Chemistry, Jadavpur University, Kolkata 700032, India; naskar.kaushik123@gmail.com (K.N.); suvendumaity99@gmail.com (S.M.); 2 Department of Chemistry, Indian Institute of Technology, Kharagpur 721302, India; himadri.maity84@gmail.com

**Keywords:** 2D Cu-MOF, square grid structure, Click reaction, Knoevenagel reaction, green chemistry

## Abstract

[Cu(CPA)(BDC)]_n_ (CPA = 4-(Chloro-phenyl)-pyridin-4-ylmethylene-amine; BDC = 1,4-benzenedicarboxylate) has been synthesized and structurally characterized by single crystal X-Ray diffraction measurement. The structural studies establish the copper (II) containing 2D sheet with (4,4) square grid structure. The square grid lengths are 10.775 and 10.769 Å. Thermal stability is assessed by TGA, and subsequent PXRD data establish the crystallinity. The surface morphology is evaluated by FE-SEM. The N_2_ adsorption−desorption analysis demonstrates the mesoporous feature (∼6.95 nm) of the Cu-MOF. This porous grid serves as heterogeneous green catalyst with superficial recyclability and thermal stability and facilitates organic transformations efficiently such as, Click and Knoevenagel reactions in the aqueous methanolic medium.

## 1. Introduction

Metal-organic frameworks (MOFs) are burgeoning targets for their potential applications in gas separation and storage [1,2,3], energy research [4,5,6,7,8,9,10], sensing of ions and molecules [11,12,13], bio-imaging [14], drug delivery [15,16], reusable and recycling sustainable catalyses [17,18], magnetism [19,20], etc. In the last few decades, such materials have received more attention towards development of more flexible catalytic materials owing to their varying symmetric and large pore volume, high surface area, tunable pore size and versatile functionality together with the diversity of metal knots and their redox states, functional groups, and the retention of crystallinity after catalytic reactions.

Zeolite imidazolate frameworks (ZIFs) [21,22,23] have arisen as a novel type of highly porous materials along with the advantages in the field of conventional MOF catalyst. By using ZIFs as solid acid catalysts, several organic transformation reactions have been carried out, such as Knoevenagel condensation [24,25], aldol condensation [26,27], Suzuki cross-coupling [28,29], Friedel–Crafts alkylation [30,31], and epoxide ring-opening reaction [32,33]. Current research in this area has been mostly focused to develop low-cost reusable recycling green catalysts to support sustainable development. Designing of multifunctional CPs or MOFs as catalyst to investigate the catalytic efficiency of various organic transformation reactions is the main focus of present research [34,35].

The copper ions play key roles in various types of biological process, such as galactose, tyrosine radical, hemocyanin, etc., also having the ability to transform various organic reactions because of its variable types of redox behaviors. The copper ion containing catalysts can be incorporated in different redox forms which may be available relatively at lower potential such as, (a) Cu(I) salts (usually in the presence of a base and/or a ligand) [36], (b) in situ reduction of Cu(II) salts (e.g., copper sulphate with sodium ascorbate or ascorbic acid) [37], (c) in situ oxidation of Cu(0) metal [38], and (d) easy comproportionation of Cu(0) and Cu(II), generally limited to special applications (e.g., biological systems) [39]. Daturi et al. demonstrated mixed valence state containing MOFs of Cu^II^/Cu^I^ ions in the HKUST-1 [40]. Moreover, Steiner and Zhang revealed that the mixed valence state Cu-MOF had dual pore size distribution and presented superior water stability compared to HKUST-1 [41]. These studies provide guidance of the catalytic activity of Cu-based MOFs. In recent times, Cu-MOFs (e.g., supported heterogeneous catalyst) have been upgraded to diminish the contamination of metal with the end product and to reuse it.

Generally, Cu(I)-catalyzed azide-alkyne cycloaddition (CuAAC) reaction, where copper present in (+1) oxidation state, is one of the best methods for the preparation of 1,4-disubstituted-1,2,3-triazoles in a regioselective manner, (as a sole regioisomer) either in aqueous or in organic medium. 1,2,3-Triazoles have received considerable interest because of their useful applications in the field of pharmaceutical agents, agrochemicals, photographic materials, etc. Moreover, in situ reduction of copper(II) complexes to copper(I) in the presence of reducing agent (e.g., sodium ascorbate, alcohols, etc.) is the most common and reliable Click reaction conditions during the assembly of diverse molecules [42]. Beside the Click chemistry, copper catalyzed Knoevenagel condensation is widely used reaction in manufacturing numerous chemical compounds which are useful for pharmaceuticals industries [43,44]. In general, this type of reaction is catalyzed by weak bases such as primary, secondary, and tertiary amines under homogeneous conditions, or it may go upward by using 40 mol% catalysts with having difficulties in catalyst recycling and recovery.

**Scheme 1:**
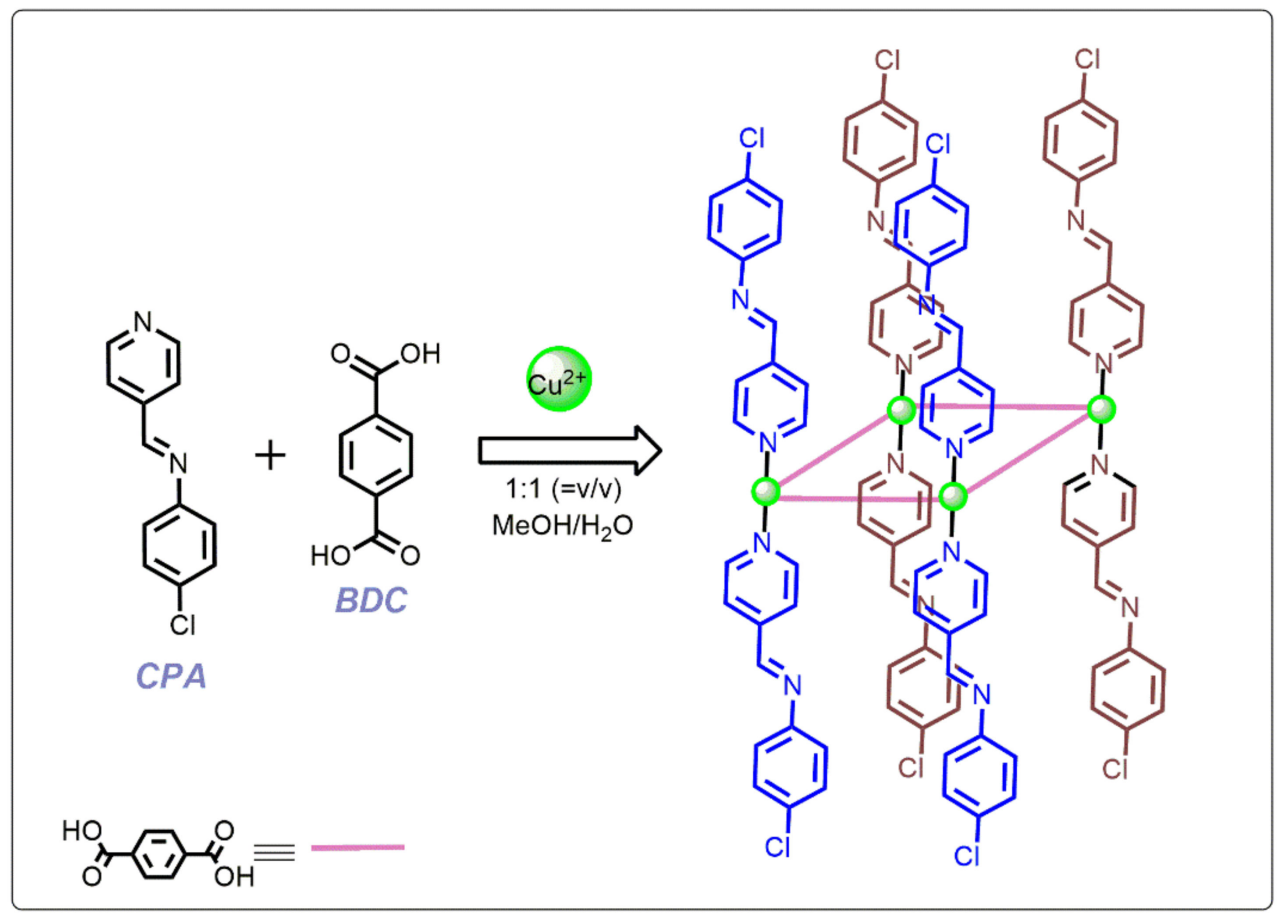
Schematic representation of synthesis of Cu-MOF (BDC = 1,4-benzenedicarboxylate).

Herein, we have successfully fabricated a mesoporous 2D Cu-MOF, [Cu(CPA)(BDC)]_n_ (CPA = 4-Chloro-phenyl)-pyridin-4-ylmethylene-amine; BDC = 1,4-benzenedicarboxylate) which can facilitate organic transformation reactions highly efficient for Click and Knoevenagel reactions in aqueous medium. The Cu-MOF was produced and well-characterized by PXRD, TGA, SXRD, SEM, BET, etc. and reusable as a heterogeneous green catalyst. Moreover, Cu-MOF offers several advantages like thermal stability and superficial recyclability in contrast with conventional catalysts (Scheme 1).

## 2. Experimental Section

### 2.1. Synthesis of (4-Chloro-phenyl)-pyridin-4-ylmethylene-amine (CPA)

In the literature, there are few reported procedures [45,46] for the CPA ligand synthesis, but herein, we have modified the synthetic procedure for better yield. A mixture of the appropriately Pyridine-4-carbaldehyde (5 mmol) and the appropriately substituted aniline (5 mmol) in dry toluene (50 mL) is refluxed for 5 h in the presence of molecular sieves (75 g; Davison, grade 514Å, effective pore size 4, 8–12 mesh beads). At the end of the reaction, the molecular sieves are filtered, washed with toluene, and the combined filtrates rotary evaporated to remove toluene. The syrupy residual material is crystallized from hexane or hexane-petroleum ether mixture to yield 97.2%, faint yellow crystalline product; IR spectrum (KBr pellet): ν_imine_ C=N, 1629; aromatic C=N, 1617; C-Cl and 687 cm^−1^ (Figure S1); ^1^H-NMR (DMSO-d_6_, 400 MHz): δ = 7.37 (2H, d, J = 8.4 Hz), 7.52 (2H, d, J = 8.4 Hz), 7.85 (2H, d, J = 4.4 Hz), 8.17 (1H, s), 8.77 (2H, d, J = 4.4 Hz) (Figure S2); ESI-MS, *m*/*z* 217.1077 [M + H]^+^ (Figure S3); elemental analysis (%) calcd for C_12_H_9_ClN_2_: C, 66.52; H, 4.19; N, 12.93. Found: 66.67; H, 4.25; N, 12.98.

### 2.2. Synthesis of Cu-MOF, [Cu(CPA)(BDC)]_n_

A solution of CPA (0.043 g, 0.2 mmol) in MeOH (5 mL) was slowly and cautiously layered to a solution of Cu(NO_3_)_2_.3H_2_O (0.048 g, 0.2 mmol), in H_2_O (5 mL) using 5 mL of 1:1 (=*v*/*v*) buffer solution of MeOH and H_2_O, followed by layering of BDC (0.033 g, 0.2 mmol) neutralizing with Et_3_N (0.021 g, 0.2 mmol) in 5 mL of EtOH. The greenish-blue-colored crystals were obtained after several days with yield 70% (0.031 g). IR spectrum (KBr pellet, cm^−1^): *ν_as_*(COO¯), 1623; *ν*_sys_(COO¯), 1392 and *ν*(C-Cl), 687 (Figure S1). Elemental analysis (%) calcd for C_20_H_12.42_ ClCuN_2_O_4_: C 54.13, H 2.82, N 6.31; found: C 54.06, H 2.95, N 6.28.

### 2.3. Crystal Structure Determination of Cu-MOF

Single dark green coloured crystals suitable for data collection obtained during synthesis were chosen under an optical microscope and mounted on glass fibres and data collection using a Bruker SMART APEX II diffractometer equipped with graphite-monochromated MoK*α* radiation (*λ* = 0.71073 Å) at 293 K. Least-squares refinements of all reflections within the hkl range −12 ≤ *h* ≤ 12, −18 ≤ *k* ≤ 18, −16 ≤ *l* ≤ 16 (**1**) used to evaluate the crystal-orientation matrices and unit cell parameters. The collected data (*I* > 2*σ*(*I*)) were integrated using the SAINT program [47], and the absorption correction was made with SADABS [48]. The molecular structure was solved using the SHELXL-2016/6 [49] package. Non-hydrogen atoms were refined with anisotropic thermal parameters. Hydrogen atoms were placed in their geometrically idealized positions and constrained to ride on their parent atoms. All calculations were carried out using the SHELXL-2016/6 [49], SHELXT 2014/4 [50], and PLATON 99 [51] programs. The crystallographic data of Cu-MOF are depicted in Table 1.

### 2.4. Characterization of MOF Catalyst

The Cu-MOF catalyst samples were measured using a Bruker D8 ADVANCE X-ray diffractometer system with Cu-K*α* radiation (*λ*  =  1.541 Å). Powder X-ray diffraction (PXRD) was executed at room temperature, and most of the PXRD patterns of as-synthesized were well-matched with the simulated patterns from single-crystal data. Thus, it signified that the bulk purity of the sample was retained. In addition, the PXRD was verified after the catalytic performance to clarifying the robustness of Cu-MOF shown in Figure S4. The thermal stability of **1** was performed within the temperature range of 32–650 °C under N_2_ atmosphere via PerkinElmer–TGA 4000 thermogravimetric analyzer and TGA analysis revealed that Cu-MOF is moderately stable up to ~360 °C (Figure S5). To authenticate, morphology of catalyst samples (before and after) was observed by using the Field Emission Scanning Electron Microscopy (FESEM; JEOL, JSM-6700F) (Figure S6).

### 2.5. Gas Adsorption Measurements

**Figure 1:**
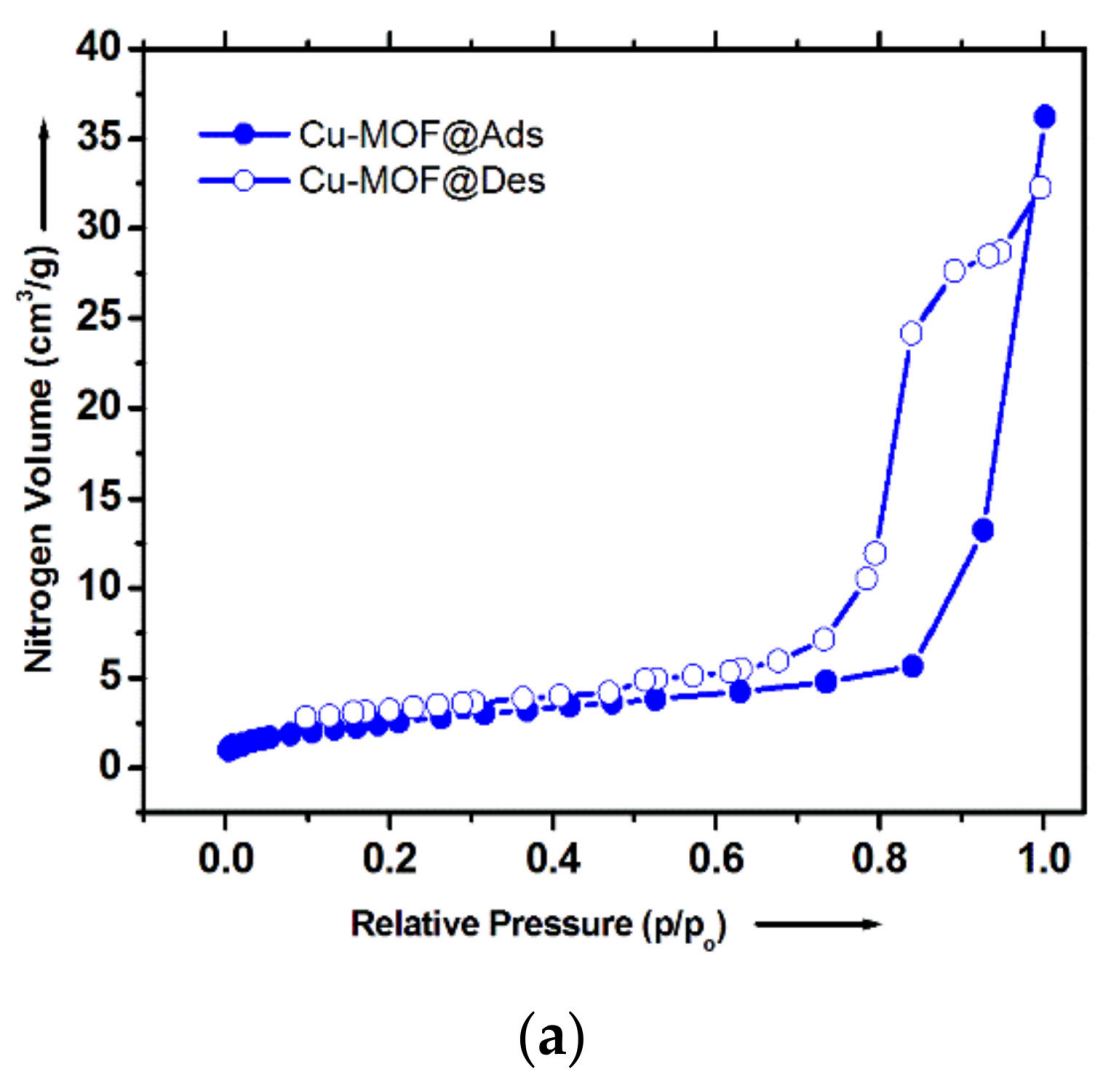

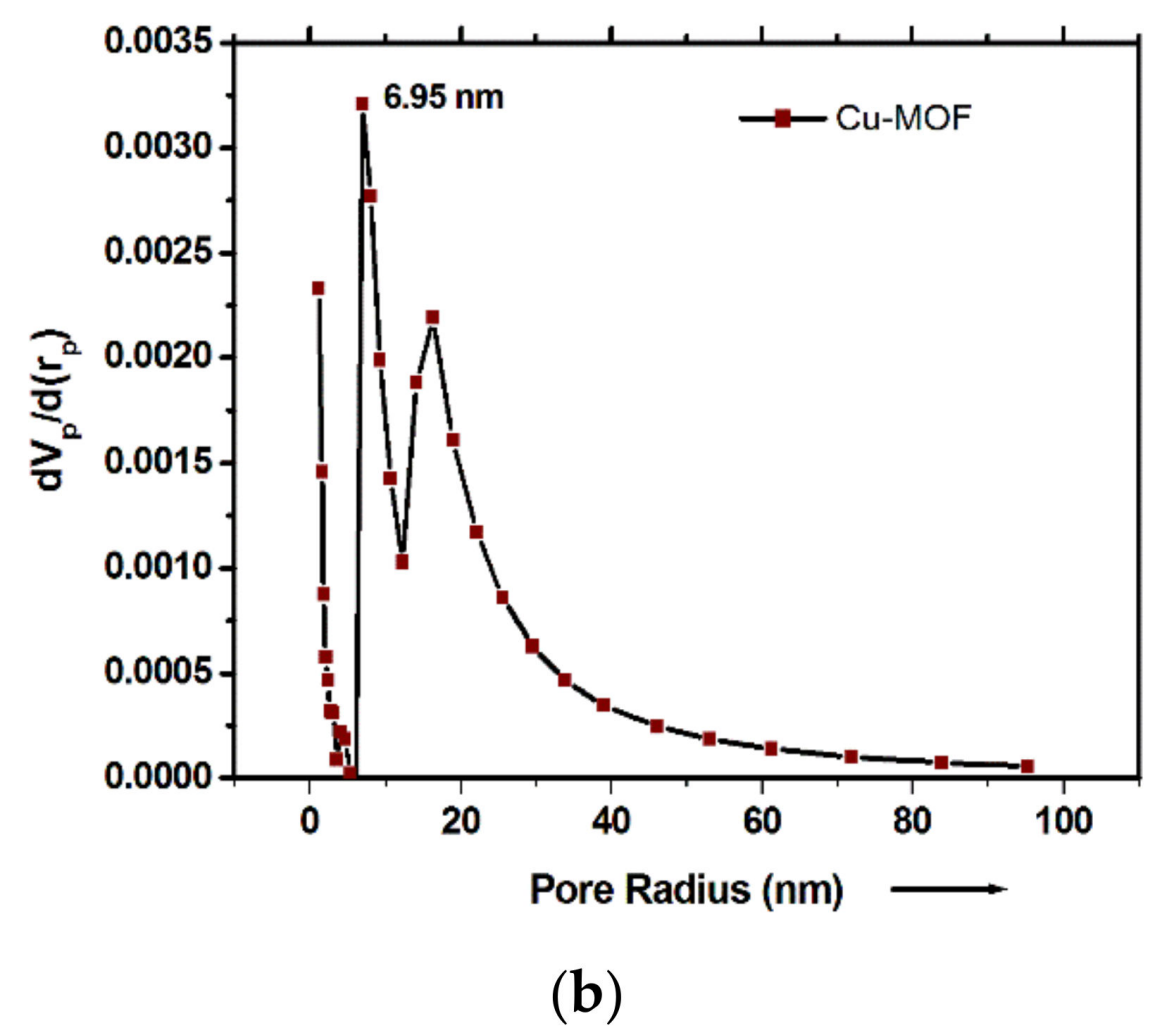
(**a**) N_2_ adsorption isotherms for Cu-MOF at 77 K and (**b**) Howarth–Kawazoe (HK) pore-size distribution calculated from N_2_ adsorption at 77 K for Cu-MOF.

The nitrogen adsorption−desorption isotherm was studied by using the dehydrated Cu-MOF sample in a Micromeritics ASAP 2420 surface area analyzer at liquid nitrogen temperature (77 K) (see Figure 1a). Prior to gas adsorption, in a tube, pretreated sample was placed and degassed for 4 h at 120 °C to remove the adsorbed solvent.

### 2.6. Catalytic Response

***Click Reaction:*** In a 50 mL round bottom flask, benzyl bromide (1 mmol), sodium azide (1.1 mmol), phenylacetylene derivatives (1 mmol), sodium ascorbate (5 mol%), K_2_CO_3_ (0.5 equivalent), and 10 mol% Cu-MOF were placed, followed by 5 mL of H_2_O-MeOH (1:4) solvent mixture. The flask was connected through a reflux condenser and heated to ~70 °C. After the completion of the reaction, the product was separated by solvent extraction process. The products were purified by column chromatography. Finally, products were characterized via ^1^H-NMR by using a 500 MHz Bruker NMR and ^13^C-NMR spectroscopy (Figure S7).

***Knoevenagel Reaction:*** A solution of different substituted benzaldehyde and active methylene compounds such as malononitrile and ethyl cyanoacetate (1:1.2, molar ratio), where the nucleophilic addition occurs by the active hydrogen compound to a carbonyl group followed by a dehydration reaction, and a molecule of water is eliminated. The product is often an α,β-unsaturated ketone in the presence of Cu-MOF (3 mol %) was stirred under room temperature conditions for 15 min (Figure S8). The solid yellow product was isolated with an excellent yield. Similarly, Knoevenagel products were also characterized through ^1^H-NMR and ^13^C-NMR spectroscopy by using a 400 MHz and 500 MHz Bruker NMR (See Figure S9). Moreover, the kinetic transformation of the reaction was analyzed through GC. The comparative catalytic activities of some MOFs for the Knoevenagel condensation reaction are summarized in Table S1.

## 3. Results and Discussion

### 3.1. Structural Descriptions of [Cu(CPA)(BDC)]_n_,

**Figure 2:**
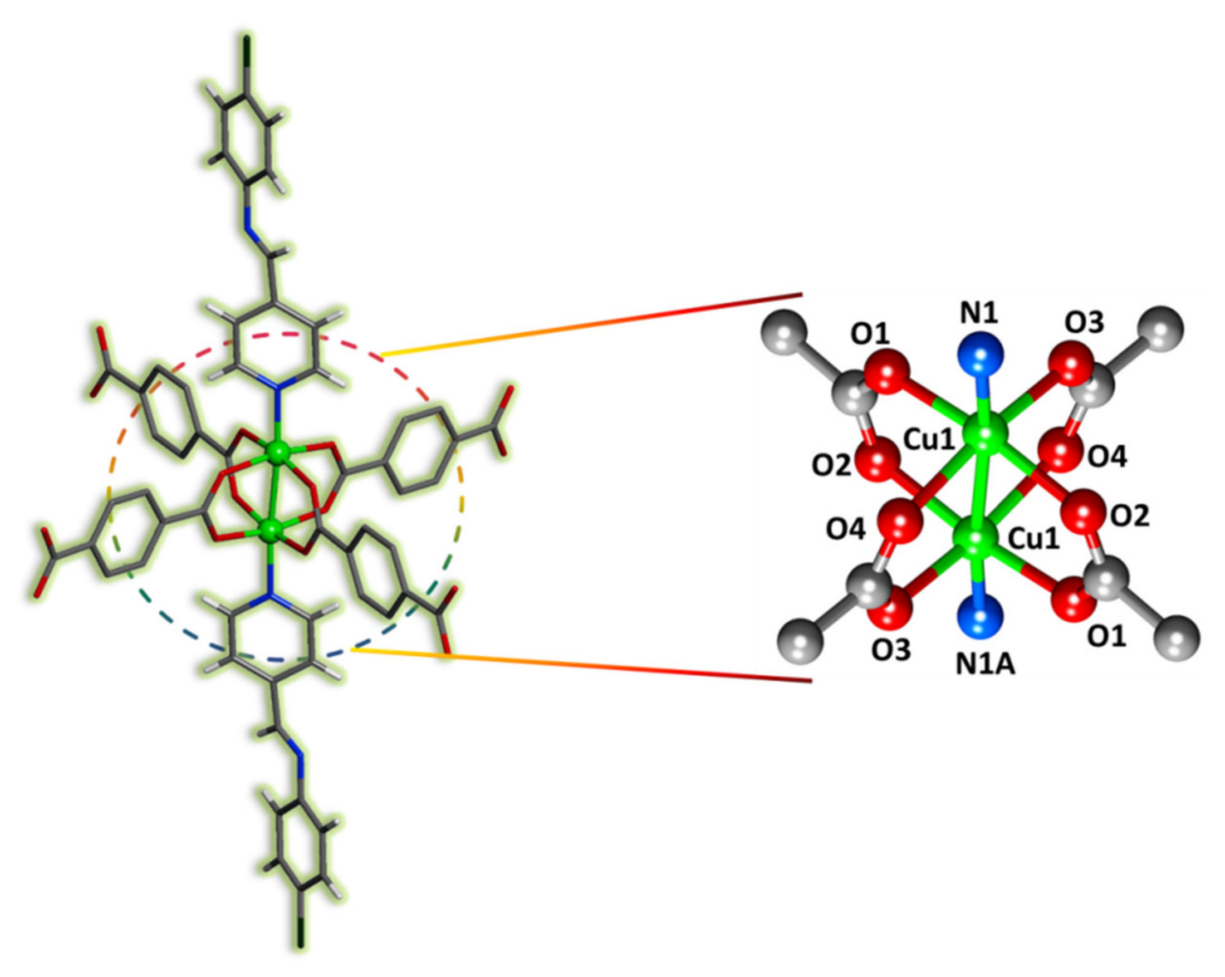
X-ray crystal structure illustrates the copper paddle-wheel secondary building unit in Cu-MOF.

The Single Crystal X-ray crystallographic analysis shows that Cu-MOF was crystallized in the monoclinic space group *P*2_1_/n with Z = 4. The asymmetric unit comprises half of the secondary building unit in Figure 2. The paddle-wheel unit of [Cu_2_(O_2_CC)_4_] was repeated along with having a crystallographic inversion center in it. In Cu-MOF, the geometry around each Cu(II) is distortional pentagonal pyramid geometry where four carboxylate groups are bridged by the BDC^2−^, and in the axial site of the Cu(II), atoms are coordinated by a CPA as a terminal ligand as shown in Figure 2.

**Figure 3:**
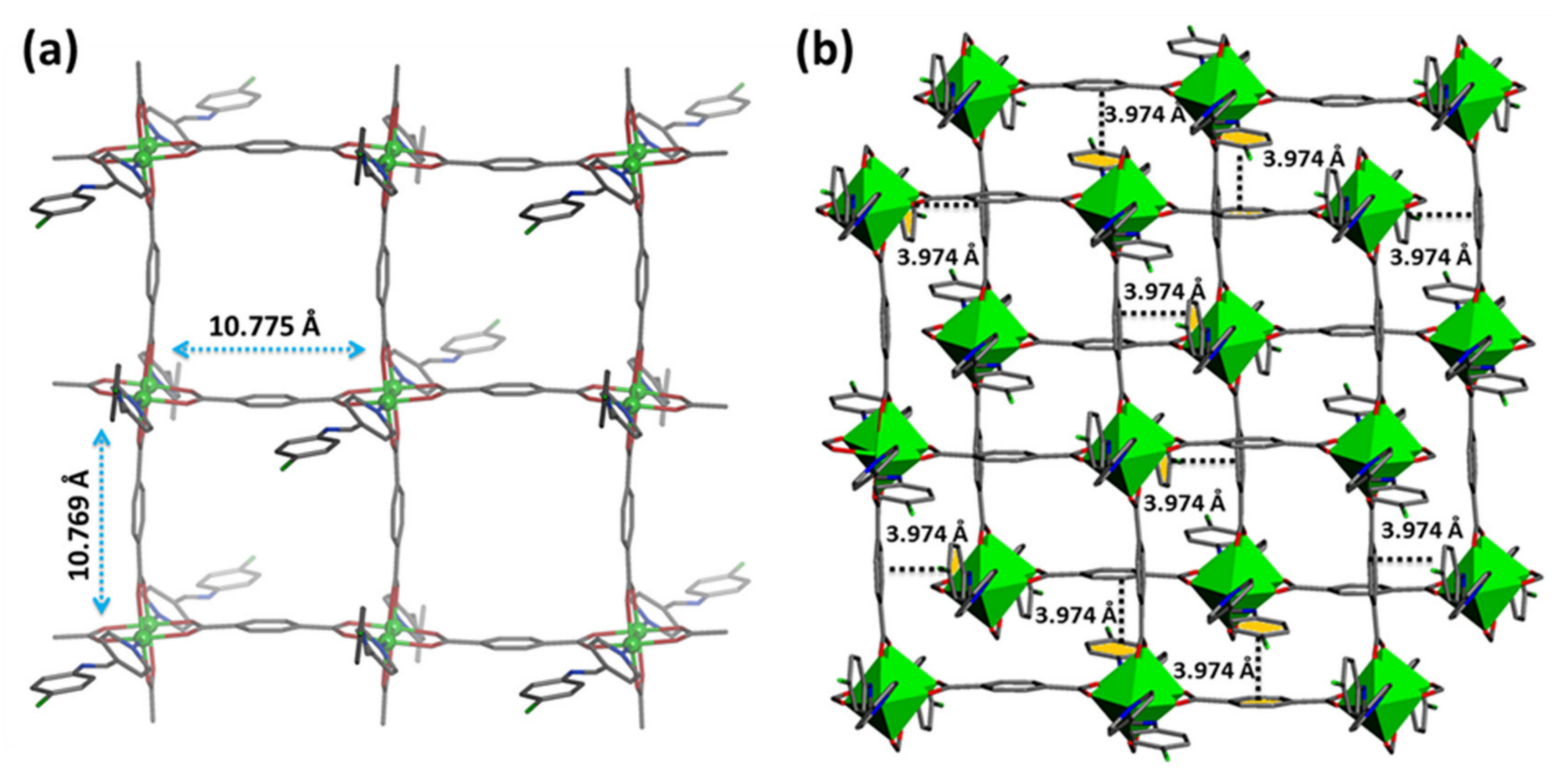
(**a**) a portion of the (4,4) net structure and (**b**) a perspective view of the 3D supramolecular aggregate of Cu-MOF by **π**–**π** interactions.

Structural motif of Cu-MOF demonstrates the coordination of BDC^2−^ to Cu(II) metal nodes as bidentate chelating mode and acts as a carboxylato-O donor to Cu(II) (Cu(1)–O(1), 1.955(3) Å; Cu(1)-O(2), 1.958(3) Å; Cu(1)-O(3), 1.961(4) Å; and Cu(1)-O(4), 1.964(3) Å) and the remaining coordinations (axial sites) are originated from the Pyridyl-N donor of CPA ligand (Cu(1)-N(1), 2.137(9) Å; Cu(1)-N(1A), 2.177(8) Å) (Table S2). The paddle-wheel units are further extended by dicarboxylate (BDC^2−^) bridged spacer ligands to construct the 2D sheet with (4, 4) square grid structure as shown in Figure 3a.

Moreover, these square grid lengths are slightly differ by 10.775 and 10.769 Å as shown in Figure 3a. The axial CPA ligands are projected on both sides of each layer. The empty voids in Cu-MOF are due to the square grids that are interlinked by **π**–**π** interactions of CPA ligands from the adjacent layers (Figure 3b). The **π**–**π** stacking distances are ~3.974 Å which are facilitated to decrease the interlayer distance between benzene rings, (CPA)^⋯^benzene rings (BDC) and thus leading to construct 3D self-assembled framework structure. The solvent-accessible void volume (246.7 Å^3^) has been estimated to be 11.5% of the total unit cell volume (2152.7 Å^3^).

The N_2_ adsorption−desorption isotherm for Cu-MOF was shown in Figure 1a at 77 K. The Cu-MOF exhibits BET specific surface area ~65.32 m^2^ g^−1^. The observed N_2_ adsorption-desorption isotherm is of type-IV category attributing to typically mesoporous structure. A hysteresis loop was observed in the relative pressure (p/p_0_) range from 0.73 to 0.99, which is of H_2_-type typically observed for different size/shape of pores inside particles. Interestingly, such hysteresis loop originated in Cu-MOF may be because of swelling of the network structure by the presence of condensed nitrogen. Again, Nitrogen adsorption/desorption Howarth–Kawazoe (HK) pore size distributions indicated the mesopores with diameter ranges from 7 to 16 nm likely due to the interparticle voids. However, the resulting **π**–**π** stacking layered structure has strengthened significant pores in the Cu-MOFs, which may allow the guest molecules to achieve the catalytic performance.

### 3.2. Catalytic Activity of Cu-MOF

#### 3.2.1. Optimization of Click Reaction

To further explore the catalytic activity of the Cu-MOF catalyst, we have chosen the synthesis of various 1,2,3 triazole derivatives catalyzed by Cu-MOF. In order to get an understanding into the optimum catalytic conditions, the reaction of sodium azide, phenylacetylene, benzyl bromide, and sodium ascorbate has been used as a model reaction. The outcome with variation of solvent, temperature, and amount of Cu-MOF catalyst on the reaction is studied in Table 2. In this reaction, the copper catalyst catalyzed azide-alkyne cycloaddition reaction via redox reaction, where Cu-MOF catalyst acts as heterogeneous catalyst. Generally, this click reaction oxidation state of copper ion changes from Cu(II) to Cu(I). [40] The similar kind of role of copper ion is observed in the catalytic reaction.

A variety of solvents are used for this reaction in the presence of 5 mol% of Cu-MOF catalyst (Table 2, entries 1–11). The yield of the reaction using H_2_O-MeOH (1:4) is maximum at 70 °C in comparison to other solvents or mixtures of solvents. After choosing H_2_O-MeOH as the solvent for the reaction, the amount of Cu-MOF catalyst and the effect of temperature are also investigated. The yield of 1-benzyl-4-phenyl-1*H*-1,2,3-triazole is 83% when the catalyst amount is 5 mol%. As evident from Table 2, on increasing the amount of catalyst to 10 mol%, the yield reaches 93%. With an increase in the amount of Cu-MOF catalyst from 10 to 15 mol%, the yield of 1-benzyl-4-phenyl-1*H*-1,2,3-triazole does not change significantly. Thus, we have performed the reaction in the presence of 10 mol% catalyst as the optimum amount. At room temperature, the yield of the product is 11% (entry 19). Increase in the reaction temperature increases the yield of the product which has been optimized at 90 °C. Even in the absence of Cu-MOF catalyst, no product is isolated (entry 13). Therefore, 10 mol% catalyst at 70 °C in H_2_O-MeOH (1:4) has been chosen under optimum conditions for the synthesis of 1,2,3-triazole derivatives.

#### 3.2.2. Substrate Scope

To investigate the generality and versatility of this method, the optimized reaction conditions are subsequently applied to the reaction of a variety of organic halides with different aliphatic and aromatic terminal alkynes (Table 3). It is observed that all reactions underwent efficiently and provided the corresponding products in good to excellent yield within a reasonable time. Electron donating and electron withdrawing substituents in the aromatic ring of the terminal alkyne do not affect significantly the product yield (Table 3, entries 2 and 4). Even the terminal alkyne containing halo-substituted aromatic ring undergoes this reaction very efficiently. The hetero-aryl substituted acetylene, 3-ethynylthiophene shows clean reaction to produce the corresponding 1-benzyl-4-(thiophen-3-yl)-1,2,3-triazole (Table 3, entry 5). Variation of halide moiety in benzyl halides shows that the benzyl bromide is more reactive than benzyl chloride under the optimized reaction conditions. According to the results précised in Table 3, the regioselectivity of the reaction is high in all cases, with only 1,4-regioisomers being produced.

The catalyst leaching has been studied as follows. The reaction is thus terminated by removal of the catalyst from the reaction mixture at half the reaction time. Residual mixture is kept separated for reaction under the same conditions. Our results indicate that the yield of product does not increase. To determine the amount of copper leaching from the catalyst, ICP-AES analysis of the liquid phase is conducted. The result of ICP-AES analysis shows no detectable amount of copper in the liquid phase. This result indicates that copper ions are not leached from the catalyst surface during the reaction.

### 3.3. Knoevenagel Condensation Catalytic Experiments

The well-known Knoevenagel condensation is a reaction between an active methylene compound and a carbonyl compound (aldehydes or ketones). This important C−C coupling reaction which is widely used in the synthesis of fine chemicals has also been investigated. The Cu-MOF plays an important role during the reaction of different substituted benzaldehyde with malononitrile and ethyl cyanoacetate (1:1.2, molar ratio) in the presence of Cu-MOF (3 mol %). As inspired by the green chemistry principles, the above reaction is needed to carry out under room temperature conditions. The catalytic transformation is executed in H_2_O:MeOH (3:1) (the mixed solvent solution) for 15 min in aerobic condition, and the solid yellow product is isolated with excellent yield (summarized in Table 4). Aliquots are withdrawn from the reaction mixture at different time intervals and thus are analyzed by GC giving kinetic data during the course of the reaction. The GC analysis also indicates that the condensation reaction has finished in 15 min. The Cu-MOF works as the heterogeneous catalysts during this organic transformation. Using this catalyst in the above reaction, the important observations are very little reaction time, room temperature, aqueous medium and high yield product. Such type of reaction can be considered as green chemistry.

### 3.4. Recyclability Cu-MOF Catalyst

**Figure 4:**
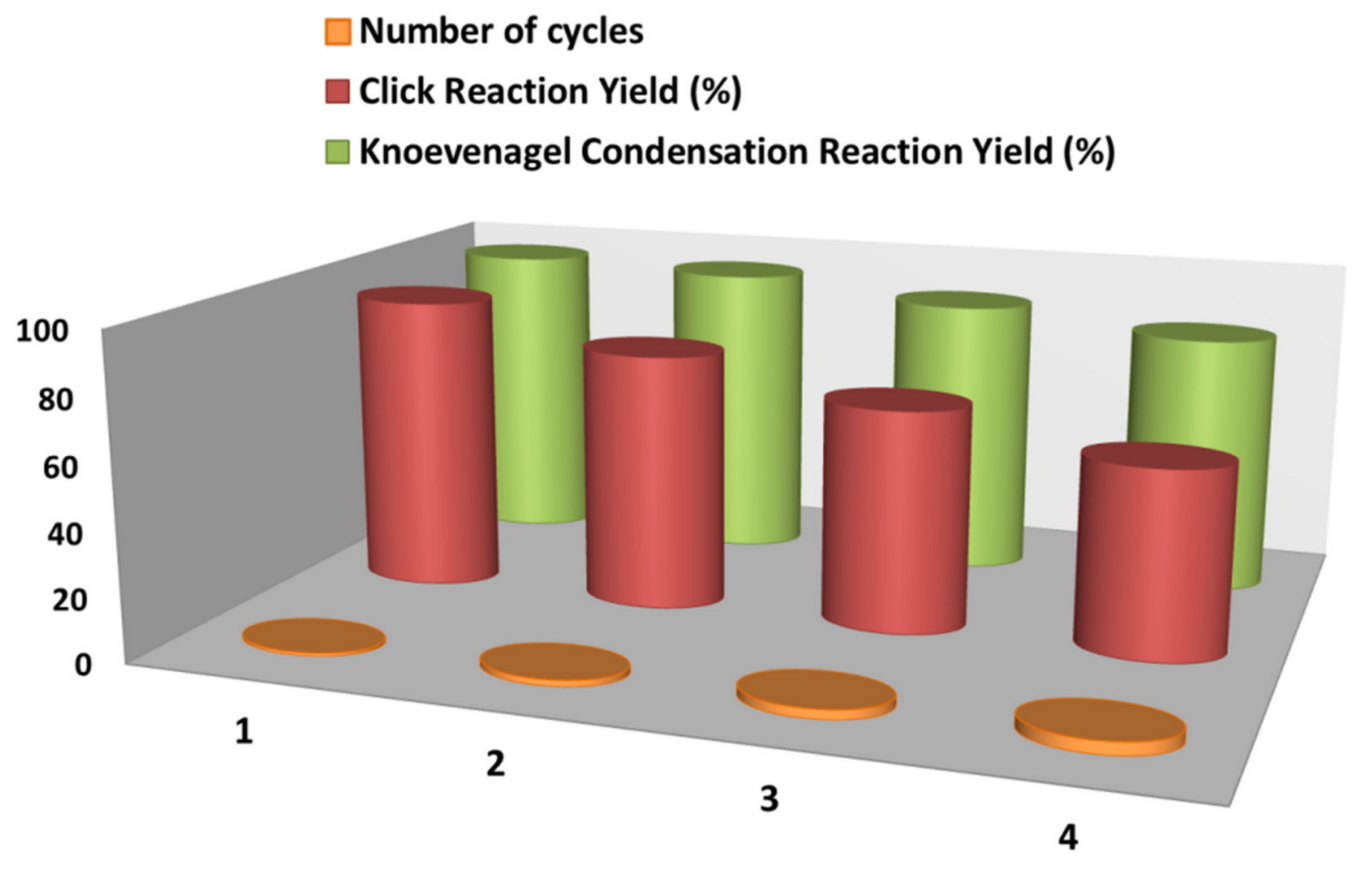
Recyclability chart of Cu-MOF upto 4th cycle.

A recovery and reusability study of the catalyst is performed for the production of 1-benzyl-4-phenyl-1*H*-1,2,3-triazole. The Cu-MOF catalyst is recovered and dried after washing with ethanol followed by acetone before reuse. We have found recyclability up to four runs although there is marginal linear loss of activity of the catalyst due to lower mass recovery in each step (Figure 4). The Au@Cu(II)-MOF effective heterogeneous catalyst for successive oxidation of alcohol and condensation reactions produce the good yield in toluene at 110 °C in air with more than 95% which is stable up to 5th cycle [52]. The other catalyst Pd(0)@UiO-68-AP which does not contain copper ion exhibits bifunctional heterogeneous catalyst for stepwise organic transformations. This promotes benzyl alcohol oxidation–Knoevenagel condensation in a stepwise way with very good yield (99%) in MeOH at room temperature [53].

The heterometallic 3D networks containing [Co^3+^–Zn^2+^] and [Co^3+^–Cd^2+^] exhibit interesting network topologies including an unprecedented one. However, these networks act as the heterogeneous and reusable catalysts for the Knoevenagel condensation reactions as well as cyanation reactions of assorted aldehydes with good yields [54]. Copper is a non noble and inexpensive abundant metal. Therefore, the use of copper ion in metal organic framework is highly encouraged to develop new catalysts for green reaction.

## 4. Conclusions

In summary, Cu-MOF catalyst acts as a highly efficient, eco-friendly, bifunctional, and heterogeneous catalytic nature. Therefore, this catalyst readily promotes Knoevenagel condensation reactions and also accelerates Click Reaction to form 1,2,3 triazole derivatives with good catalytic activity as well as excellent conversions even in water and MeOH at room temperature using a low amount of catalyst and a short reaction time. The porous square grid structure of Cu-MOF performs this bifunctional activity in solid catalysts for a broad scope of organic reactions.

## Figures and Tables

**Table 1:** Crystal data and refinement parameters for Cu-MOF.

CCDC No.	2094389
formula	C_20_H_12.4_ ClCuN_2_O_4_
formula weight	443.74
crystal system	monoclinic
space group	*P*2_1_/n
a (Å)	10.2699 (4)
b (Å)	15.3188 (6)
c (Å)	14.1931 (6)
*β* (°)	105.404 (2)
V (Å^3^)	2152.68 (15)
T (K)	293 (2)
Z	4
D_calcd_ (g/cm^3^)	1.369
*µ* (mm^−1)^	1.164
*λ*(Å)	0.71073
*θ* range (°)	2.98–25.01
total reflections	3795
unique reflections	2336
refined parameters	296
R_1_^a^ [*I* > 2*σ*(*I*)]	0.0533
*w*R_2_^b^	0.1569
Goodness-of-fit	1.008
difference between peak and hole (e·Å^−3^)	0.595−0.443

^a^ R_1_ = Σ||*F*_o_| − |*F*_c_||/Σ|*F*_o_|, ^b^ *w*R_2_ = [Σ*w*(*F*_o_^2^ − *F*_c_^2^)^2^/Σ*w*(*F*_o_^2^)^2^]/2, for 1, *w* = 1/[*σ*^2^(*F*_o_^2^) + (0.0771P)^2^+ 3.2709P], where P = (*F*_o_^2^ + 2*F*_c_^2^)/3.

**Table 2:**
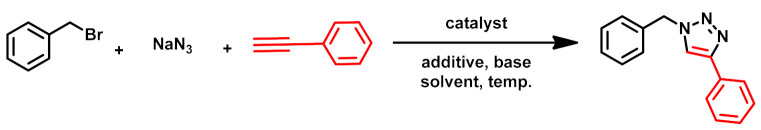
Optimization of reaction conditions ^a^.

Entry	Catalyst	Amount of Catalyst	Solvent	Time (h)	T (°C)	Yield (%) ^b^
1	Cu-MOF	5 mol%	Neat	7	70	trace
2	Cu-MOF	5 mol%	DMF	7	70	42
3	Cu-MOF	5 mol%	THF	7	70	43
4	Cu-MOF	5 mol%	CH_3_CN	7	70	48
5	Cu-MOF	5 mol%	H_2_O	7	70	68
6	Cu-MOF	5 mol%	Toluene	7	70	29
7	Cu-MOF	5 mol%	Ethanol	7	70	64
8	Cu-MOF	5 mol%	Methanol	7	70	59
9	Cu-MOF	5 mol%	H_2_O-MeOH	7	70	83
10	Cu-MOF	5 mol%	H_2_O-THF	7	70	49
11	Cu-MOF	5 mol%	H_2_O-DMF	7	70	55
12	Cu-MOF	5 mol%	H_2_O-MeOH	9	70	39
13	---	-	H_2_O-MeOH	12	80	--
14	Cu-MOF	3 mol%	H_2_O-MeOH	7	70	54
15	Cu-MOF	5 mol%	H_2_O-MeOH	7	70 ^c^	83
16	Cu-MOF	5 mol%	H_2_O-MeOH	7	70 ^d^	22
17	Cu-MOF	10 mol%	H_2_O-MeOH	7	70	93
18	Cu-MOF	15 mol%	H_2_O-MeOH	7	70	93
19	Cu-MOF	10 mol%	H_2_O-MeOH	7	rt	11
20	Cu-MOF	10 mol%	H_2_O-MeOH	7	90	93
21	Cu-MOF	10 mol%	H_2_O-MeOH	7	70 ^e^	93
22	Cu-MOF	10 mol%	H_2_O-MeOH	7	70 ^f^	57

^a^ Reaction conditions: phenylacetylene (1 mmol), benzyl bromide (1 mmol), sodium azide (1.1 mmol), sodium ascorbate (5 mol%), K_2_CO_3_ (0.5 equivalent), solvent (5 mL). ^b^ Isolated yield. ^c^ 10 mol% sodium ascorbate. ^d^ Without sodium ascorbate. ^e^ K_2_CO_3_ (1 equivalent), ^f^ Without K_2_CO_3_.

**Table 3:**
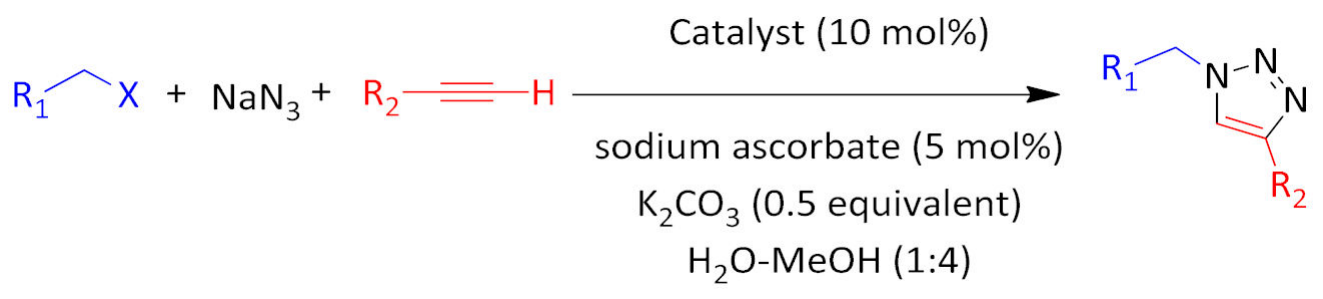
Cu-MOF catalyzed one-pot azide preparation from organic halide followed by cycloaddition with terminal alkyne.

Entry	Aliphatic Halide	Alkyne	Triazole	Yield (%) ^a^
**1**	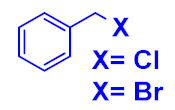	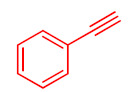	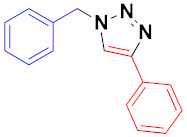	84 93
**2**	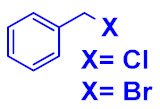	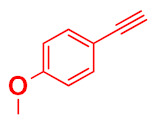	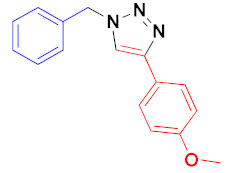	81 89
**3**	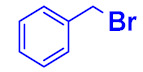	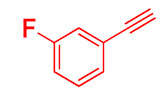	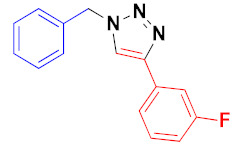	78
**4**	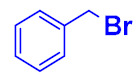	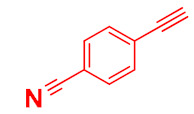	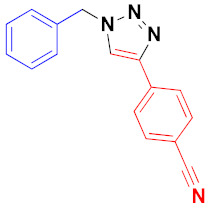	80
**5**	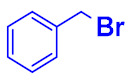	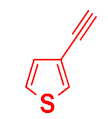	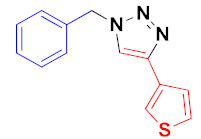	82

^a^**Reaction conditions:** phenylacetylene (1 mmol), benzyl bromide (1 mmol), sodium azide (1.1 mmol), sodium ascorbate (5 mol%), K_2_CO_3_ (0.5 equivalent), H_2_O-MeOH (5 mL).

**Table 4:**
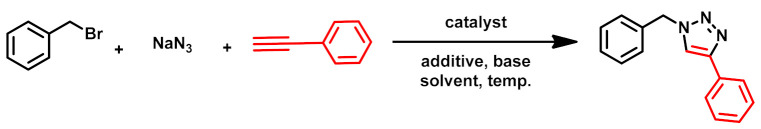
Knoevenagel condensation catalytic experiments.

Entry	R_1_	Time	Product	Conversion (%)
1.	-OH	15 min	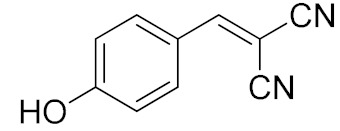	>99
2.	-OH	15 min	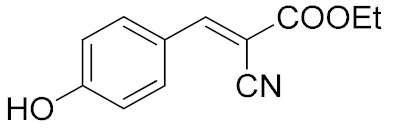	>98
3.	-OMe	15 min	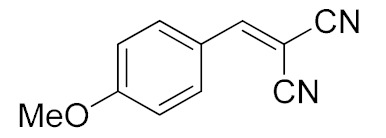	>99
4.	-OMe	15 min	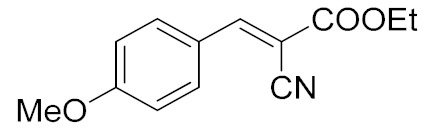	>97
5.	-OMe, -OH	15 min	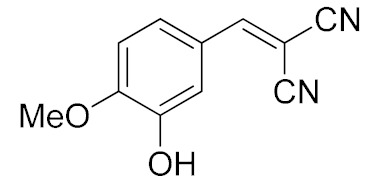	>99
6.	-OMe, -OH	15 min	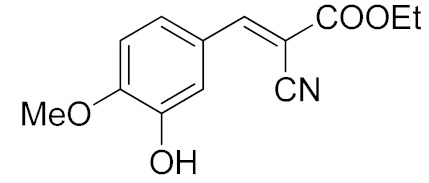	>99
7.	-OMe, -OH	15 min	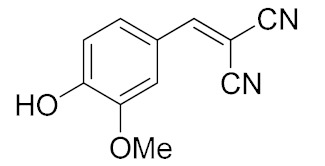	>99
8.	-OMe, -OH	15 min	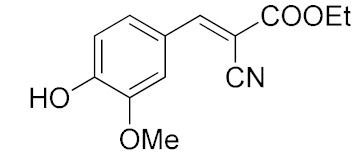	>98

## Data Availability

Not applicable.

## Supplementary Materials

The following are available online. Figure S1: IR spectrum of CPA and Cu-MOF (1). Figure S2: ^1^H-NMR of CPA. Figure S3: ESI-MS of CPA. Figure S4: PXRD plots of Cu-MOF. Figure S5: Thermogravimetric analysis plots of Cu-MOF. Figure S6: FE-SEM image of crystalline morphologies of Cu-MOF. Table S1: The catalytic activity of Some MOFs in the Knoevenagel condensation reaction. Table S2: List of selective bond lengths and bond angles of Cu-MOF, List of ^1^H-NMR and ^13^C-NMR spectroscopy of Click Reactions (Figure S7). Figure S8: Solvent dependent catalytic assay of Knoevenagel Reaction. Figure S9: List of ^1^H-NMR and ^13^C-NMR spectroscopy of Knoevenagel Reactions.

## Sample Availability

Samples of the compounds are not available from the authors.
