# Supplementary material for: A Reusable Efficient Green Catalyst of 2D Cu-MOF for the Click and Knoevenagel Reaction
Source: Molecules. 2021 Aug 31;26(17):5296. doi: 10.3390/molecules26175296 (PMC8434330; doi:10.3390/molecules26175296)
Supplement: Supplementary file 1 [file molecules-26-05296-s001.zip › molecules-1337207-supplementary.pdf]

Supporting Information

A Reusable Efficient Green Catalyst of 2D Cu-MOF for the Click and Knoevenagel Reaction

Kaushik Naskar,[a] Suvendu Maity,[a] Himadri Sekhar Maity,[b] and Chittaranjan Sinha[, a]*

[a]Department of Chemistry, Jadavpur University, Kolkata–700032, India.

[b]Department of Chemistry, Indian Institute of Technology, Kharagpur, India.

Sl. No.	Contents	Page
1	IR spectrum of CPA and Cu-MOF (1) (Figure S1)	S2
2	^{1}H NMR of CPA (Figure S2)	S2
3	ESI-MS of CPA (Figure S3)	S3
4	PXRD plots of Cu-MOF **(1)** (Figure S4)	S3
5	Thermogavimetric analysis plots of Compound **1 (**Figure S5)	S4
6	FE-SEM image of crystalline morphologies of Cu-MOF (Figure S6)	S4
7	The catalytic activity of Some MOFs in the Knoevenagel condensation reaction (Table S1)	S5
8	List of selective bond lengths and bond angles of **1** (Table S2)	S5
9	List of ^{1}H-NMR and ^{13}C-NMR spectroscopy of Click Reactions (Figure S7)	S6-S10
10	Solvent dependent catalytic assay of Knoevenagel Reaction (Figure S8)	S11
11	List of ^{1}H-NMR and ^{13}C-NMR spectroscopy of Knoevenagel Reactions (Figure S9)	S11-S19

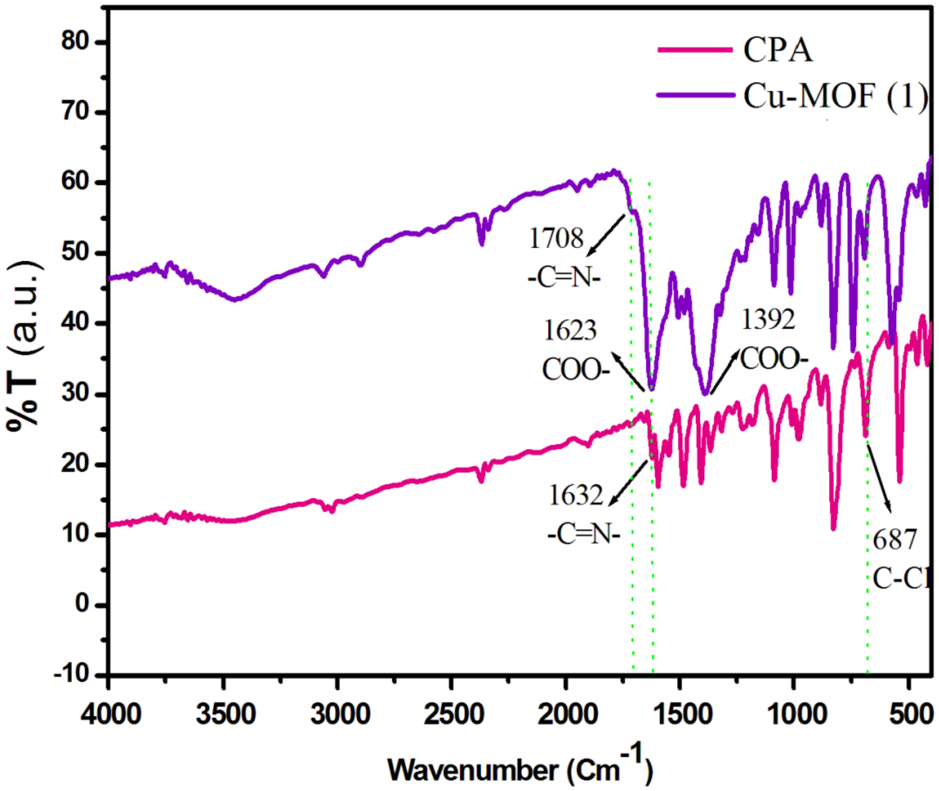

Figure S1. IR spectrum of CPA and Cu-MOF (1).

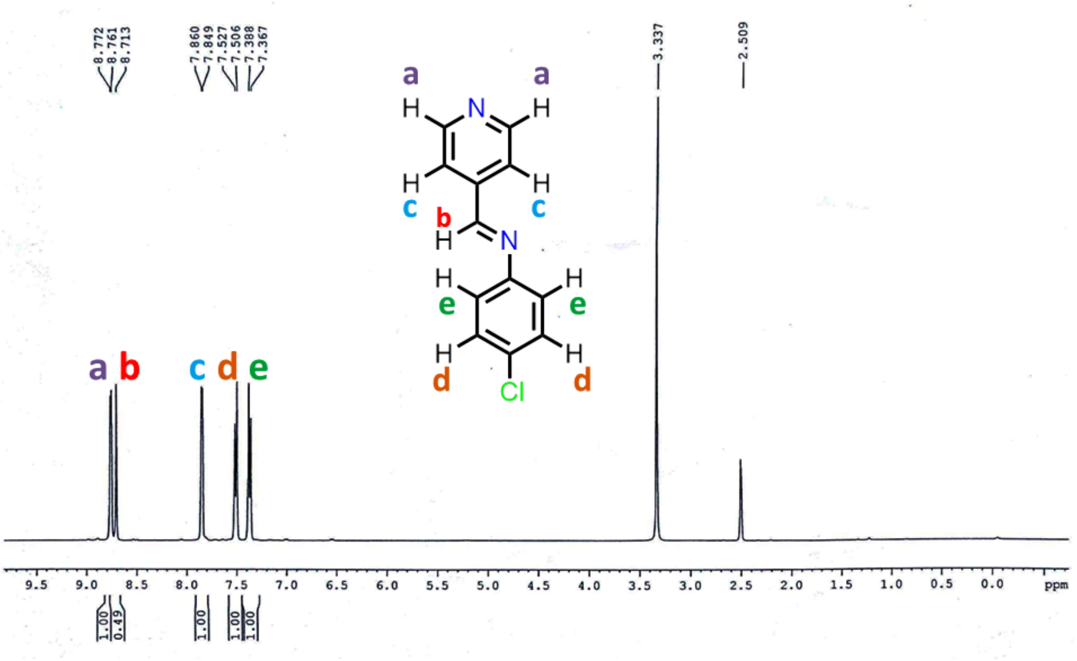

Figure S2. ^{1}H NMR of **CPA** (DMSO-d6, 400 MHz): δ = 7.37 (2H, d, J = 8.4 Hz), 7.52 (2H, d, J = 8.4 Hz), 7.85 (2H, d, J = 4.4 Hz), 8.17 (1H, s), 8.77 (2H, d, J = 4.4 Hz);

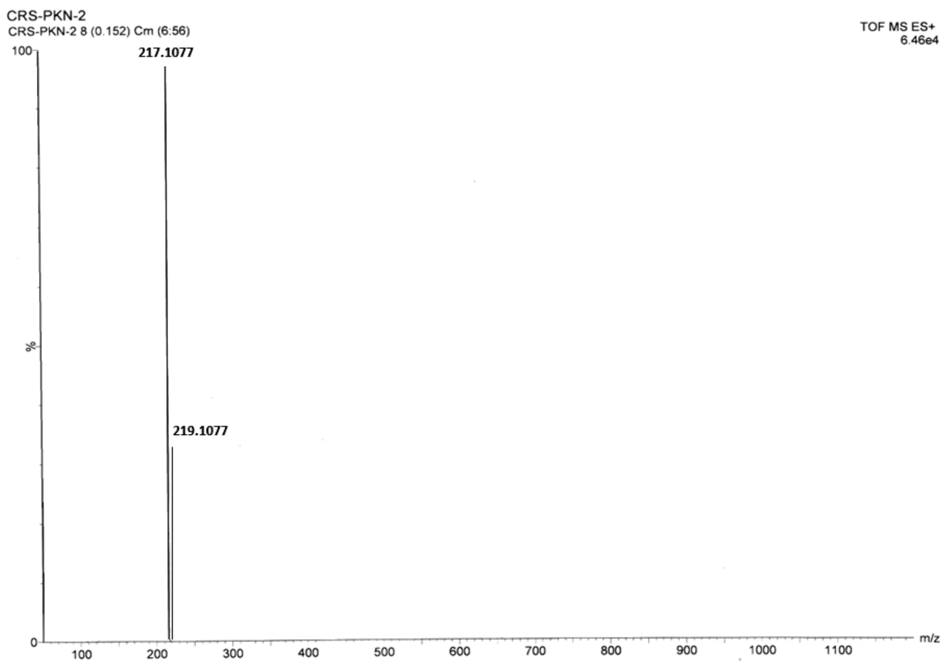

Figure S3. ESI-MS of **CPA**, m/z 217.1077 $[M+H]^+$.

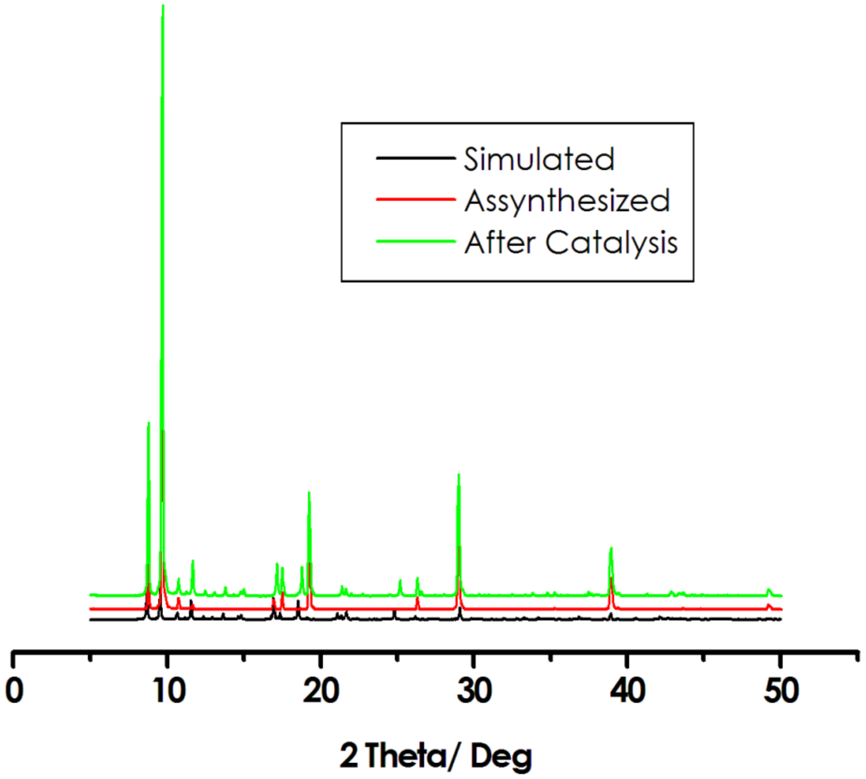

Figure S4. PXRD plots of Cu-MOF **(1)** simulated (black), assynthesized (red) and after catalysis (green).

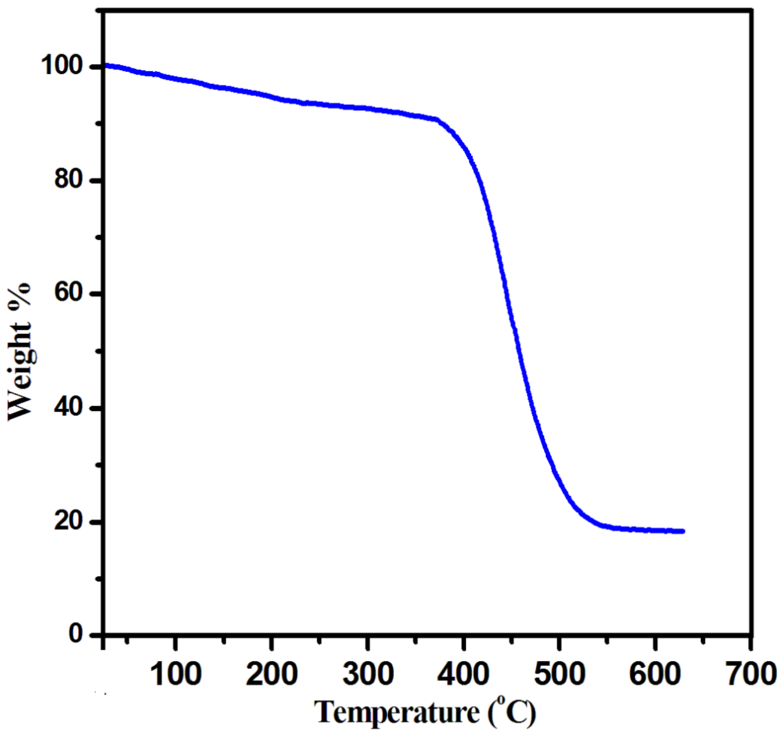

Figure S5. Thermogavimetric analysis plots of Compound **1**.

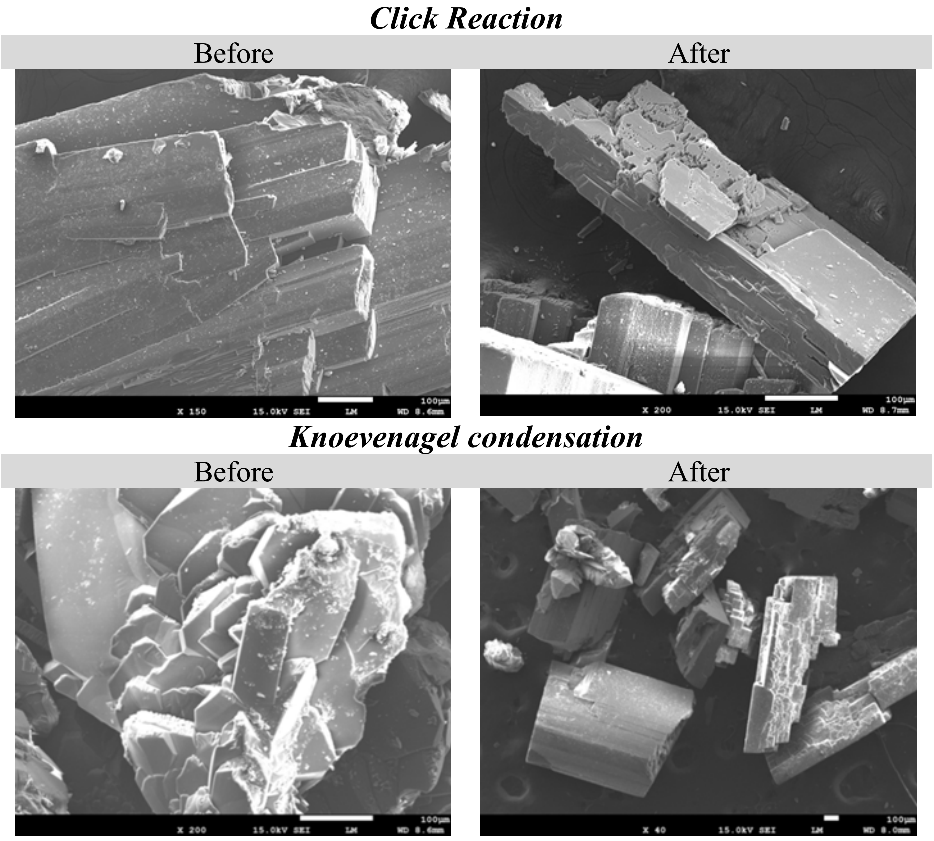

Figure S6. FE-SEM image of crystalline morphologies of Cu-MOF before and after catalytic experiments.

Table S1. The catalytic activity of Some MOFs in the Knoevenagel condensation reaction

**Sl. No.**	**MOFs/CPs Based Catalysts**	**Solvent**	**Temp. (°C)**	**Time (h)**	**Conversion (%)**	**Ref.**
Benzaldehyde with Ethyl cyanoacetate						
1.	UiO-66-NH_2	Ethanol	80	2	94	1
2.	Mg_2dobdc	Toluene	70	4	85	2
3.	$Ba_2(BTC)(NO_3)$	Toluene	110	24	14	3
4.	Mg-ABDC	Ethanol	80	7	~95	4
5.	Cu-MOF	H_2O:MeOH (3:1)	RT	0.25	~98	This Work
Benzaldehyde with Malononitrile						
6.	$Pb(cpna)_2 \cdot 2DMF \cdot 6H_2O$ (3 mol%)	CH_3CN	R.T.	24 h	100	5
7.	$[Cd(4btapa)_2(NO_3)_2] \cdot 6H_2O \cdot 2DMF$ (5 mol%)	C_6H_6	R.T.	12 h	98	6
8.	$[Cd(bipd)_2(DMF)_2] \cdot (ClO_4)_2 \cdot (2DMF)$ (4 mol%)	C_6H_6	R.T.	30 min	93	7
9.	$[Gd_2(tnbd)_3(DMF)_4] \cdot 4DMF \cdot 3H_2O$ (10 mol%)	C_6H_6	R.T.	20 min	96	8
10.	Au@Cu(II)-MOF (1) (3 mol%)	Toluene/MeOH	R.T.	23 h	99	9
11.	$\{[Ni_3(TBIB)_2(BTC)_2(H_2O)_6] \cdot 5C_2H_5OH \cdot 9H_2O\}_n$ (5 mol%)	DCM	60 °C	2 h	100	10
12.	TMU-55 (0.5 mol%)	H_2O	R.T.	5 min	99	11
13.	HTMU-55 (0.5 mol%)	H_2O	R.T.	5 min	87	11
14.	Fe_3O_4@ZIF-8 (4 mol%)	Toluene	R.T.	3 h	94	12

Table S2. List of selective bond lengths and bond angles of **1**.

**Compound 1**			
**Bond length**	**(Å)**	**Angle**	**Deg (°)**
Cu1-O1	1.955(3)	∠O1-Cu1-O2	167.25(13)
Cu1-N1A	2.177(8)	∠O1-Cu1-O3	89.92(16)
Cu1-N1	2.137(9)	∠O2-Cu1-O3	88.65(16)
Cu1-O4	1.964(3)	∠O1-Cu1-O4	89.48(16)
Cu1-O2	1.958(3)	∠O2-Cu1- O4	89.14(16)
Cu1- O3	1.961(4)	∠O3- Cu1-O4	167.33(13)
Cu1-Cu1	2.6633(9)	∠O1-Cu1-N1	95.5(3)
		∠O2-Cu1-N1	97.2(3)
		∠O3-Cu1-N1	100.0(3)
		∠O4-Cu1-N1	92.7(3)

Figure S7. List of ^{1}H-NMR and ^{13}C-NMR spectroscopy of Click Reactions**.**

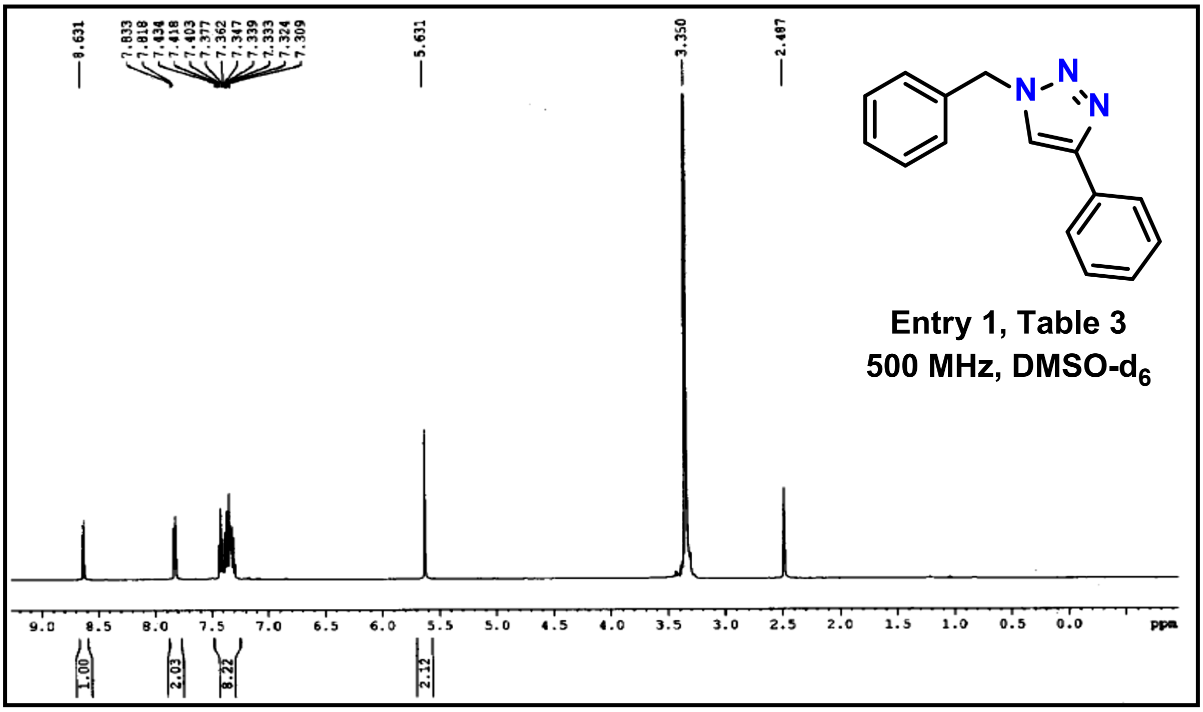

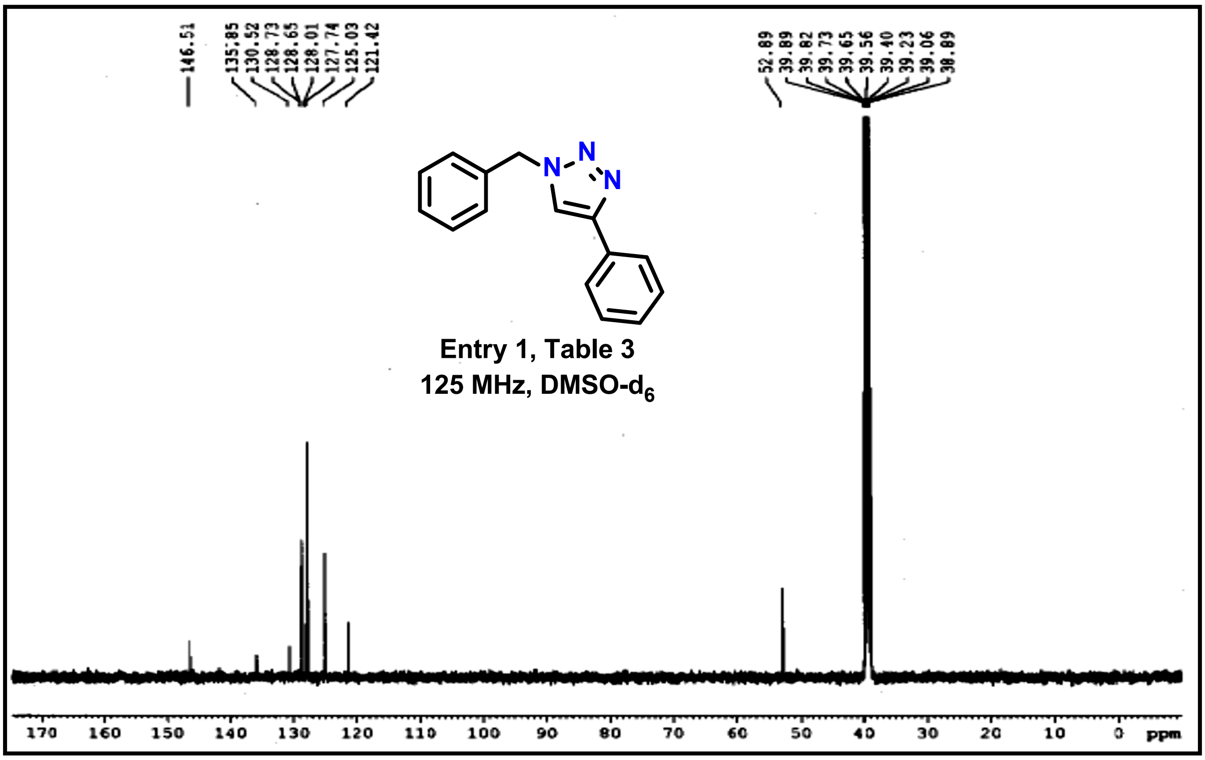

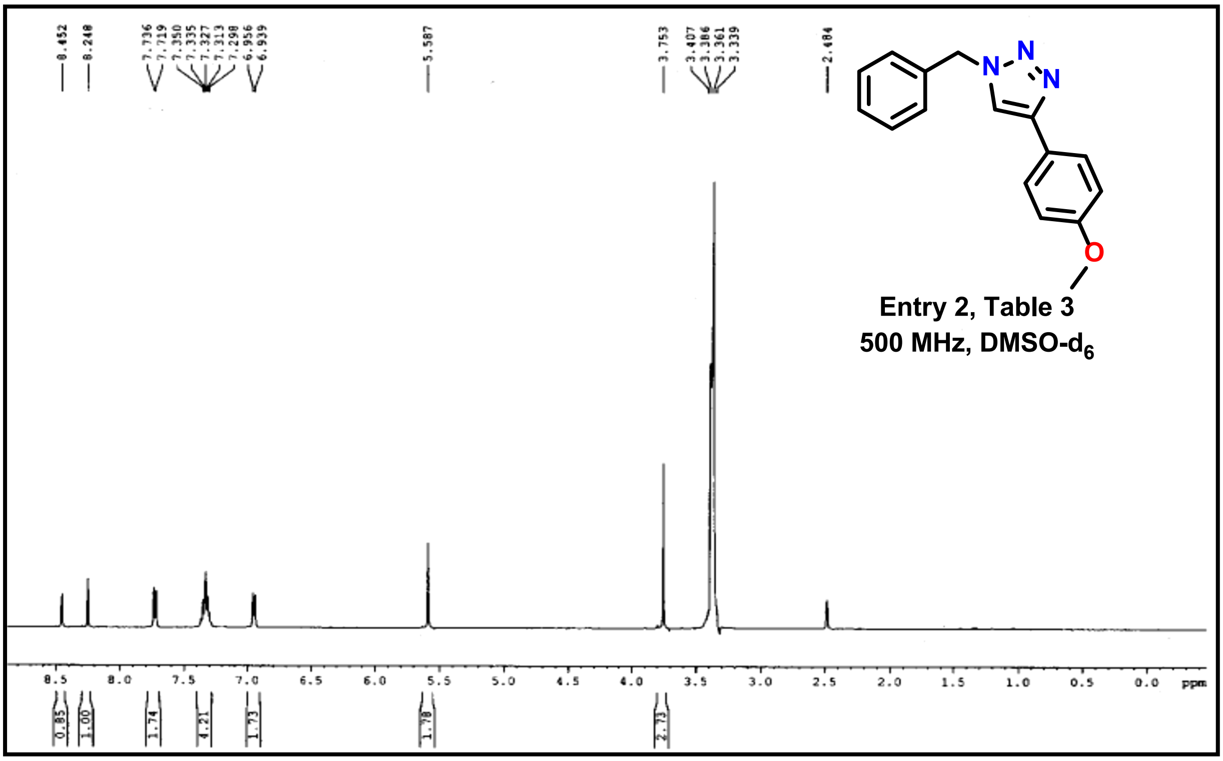
8.452
8.248
7.736
7.719
7.350
7.335
7.327
7.313
7.298
6.956
6.939
5.587
3.753
3.407
3.386
3.361
3.339
2.484
Entry 2, Table 3
500 MHz, DMSO-d6
ppm
0.85
1.00
1.74
4.21
1.73
1.78
2.73

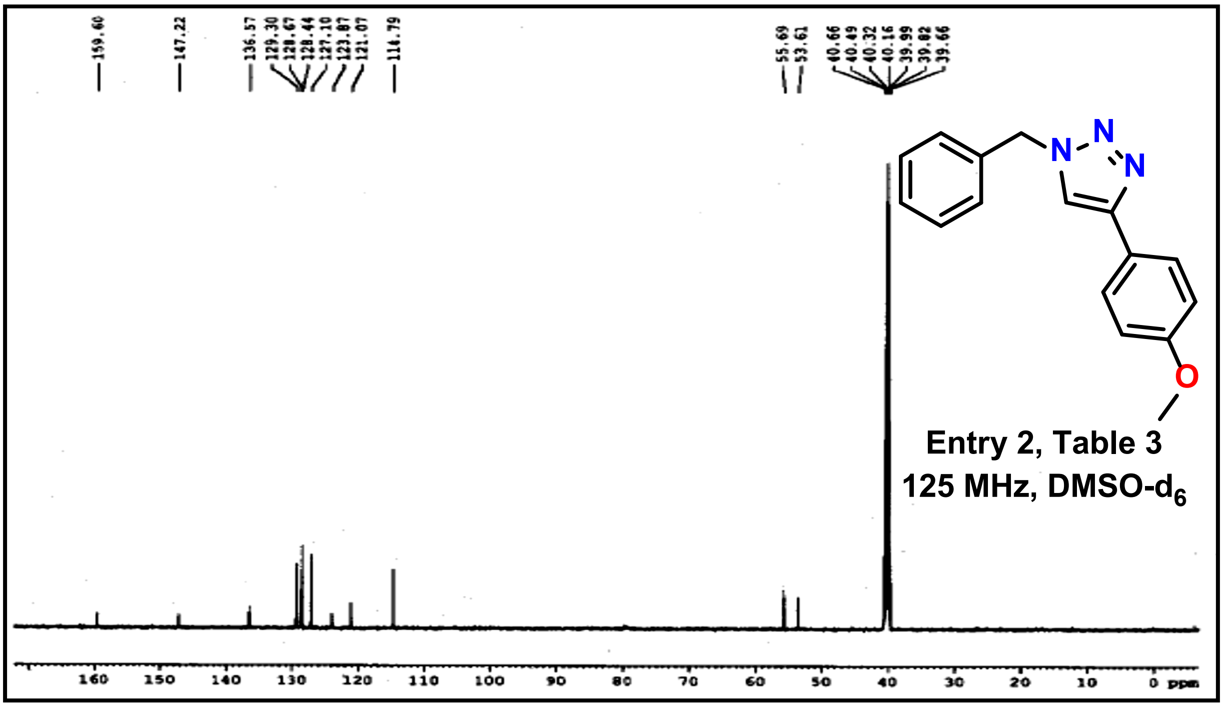
159.60
147.22
136.57
129.30
128.67
128.44
127.10
123.87
121.07
114.79
55.69
53.61
40.66
40.49
40.32
40.16
39.99
39.82
39.66
Entry 2, Table 3
125 MHz, DMSO-d6
ppm

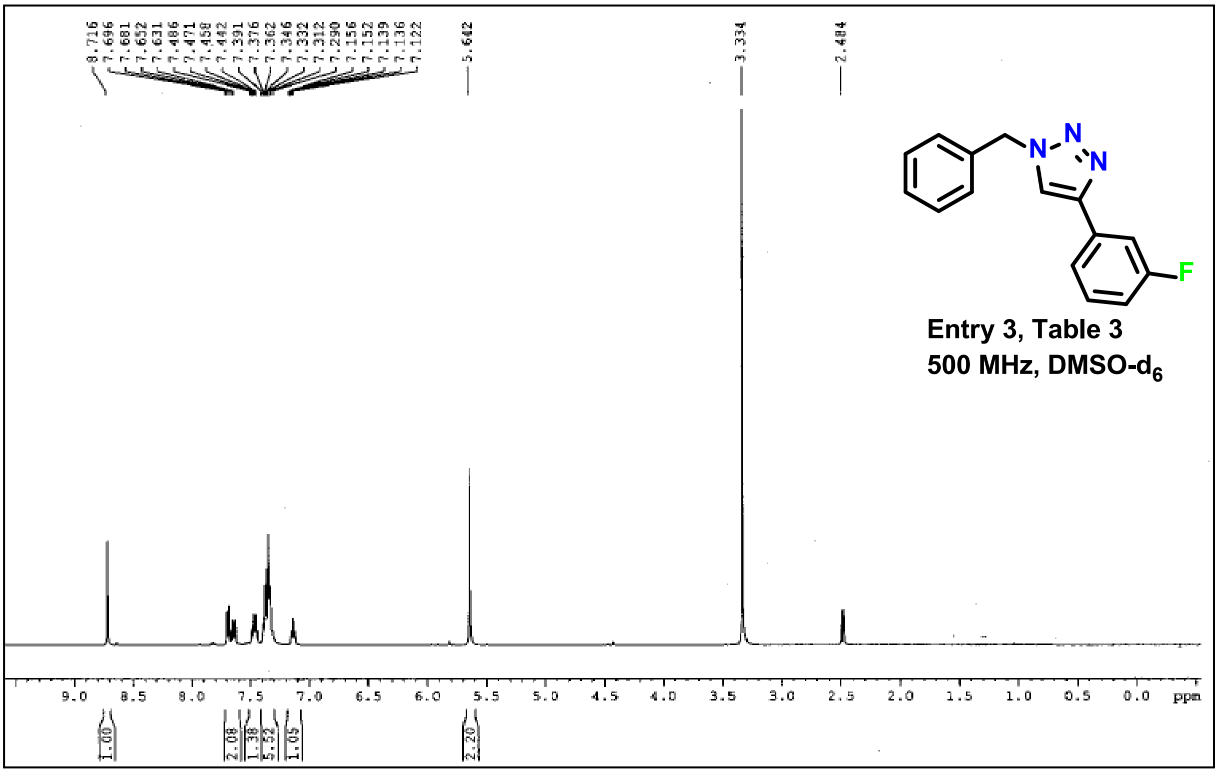

8.716
7.696
7.681
7.652
7.631
7.486
7.471
7.458
7.442
7.391
7.376
7.362
7.346
7.332
7.312
7.290
7.156
7.152
7.139
7.136
7.122
5.642
3.334
2.484
N
N
N
F
Entry 3, Table 3
500 MHz, DMSO-d6
9.0 8.5 8.0 7.5 7.0 6.5 6.0 5.5 5.0 4.5 4.0 3.5 3.0 2.5 2.0 1.5 1.0 0.5 0.0 ppm
1.00
2.08
1.38
5.52
1.05
2.20

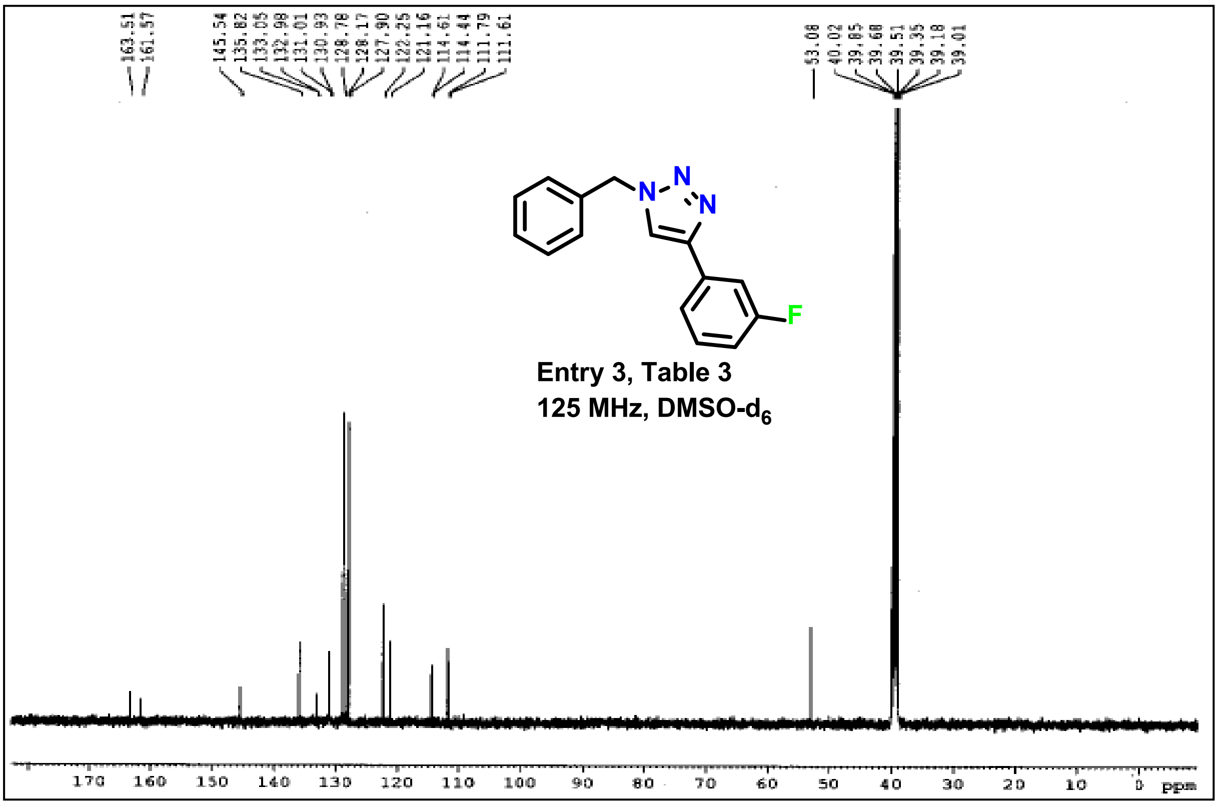

163.51
161.57
145.54
135.82
133.05
132.98
131.01
130.93
128.78
128.17
127.90
122.25
121.16
114.61
114.44
111.79
111.61
53.08
40.02
39.85
39.68
39.51
39.35
39.18
39.01
N
N
N
F
Entry 3, Table 3
125 MHz, DMSO-d6
170 160 150 140 130 120 110 100 90 80 70 60 50 40 30 20 10 0 ppm

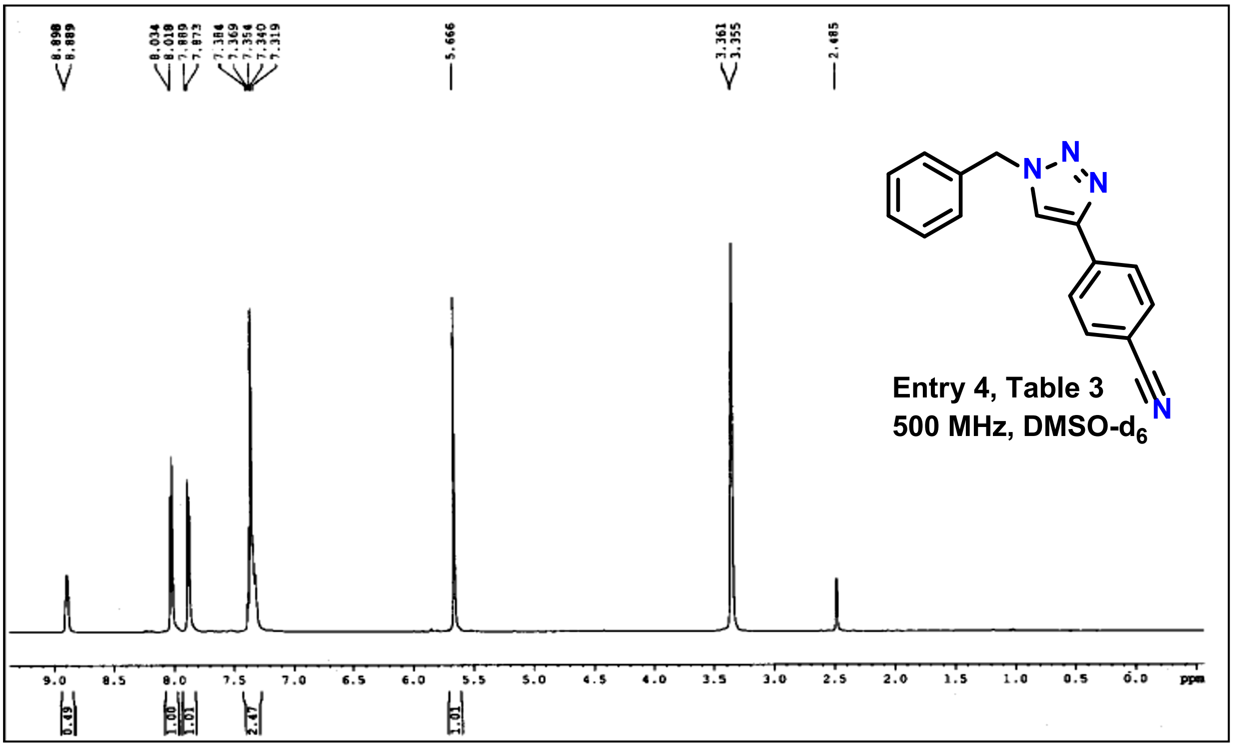

Entry 4, Table 3
500 MHz, DMSO-d6
8.898
8.889
8.034
8.018
7.889
7.873
7.384
7.369
7.354
7.340
7.319
5.666
3.361
3.355
2.485
ppm
0.49
1.00
1.01
2.47
1.01

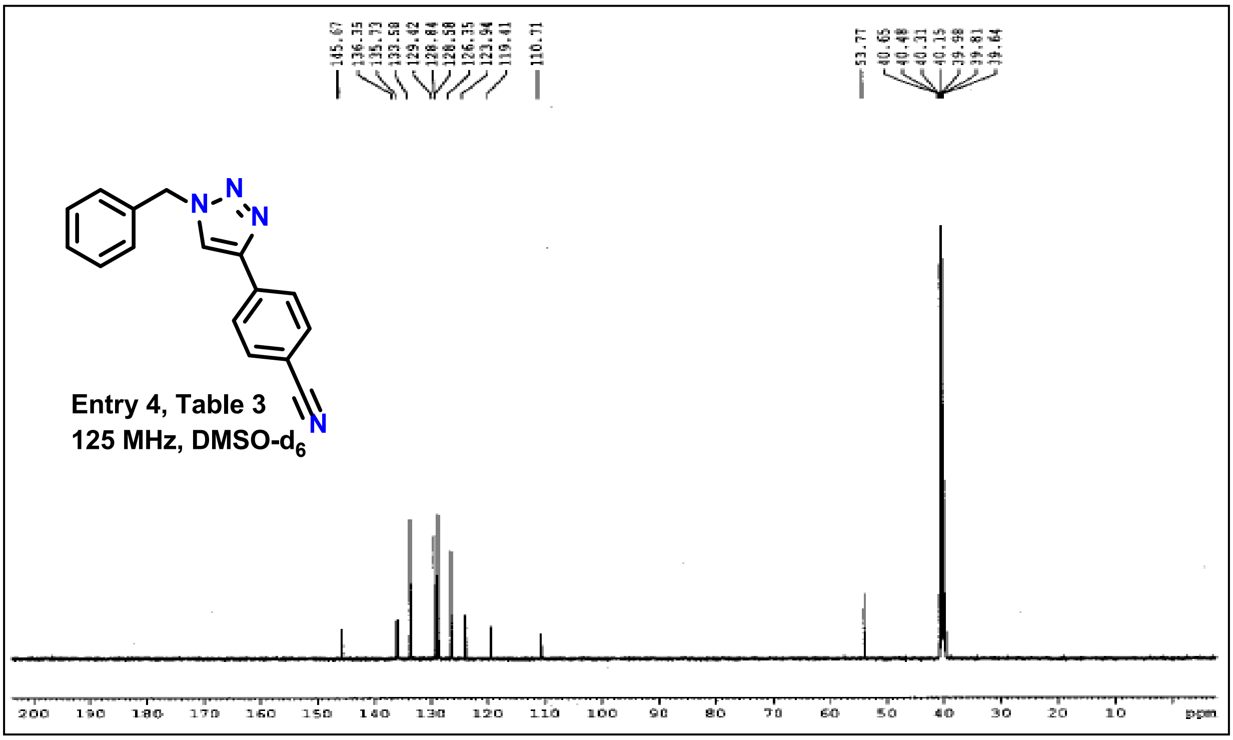

Entry 4, Table 3
125 MHz, DMSO-d6
145.67
136.35
135.73
133.58
129.42
128.84
128.58
126.35
123.94
119.41
110.71
53.77
40.65
40.48
40.31
40.15
39.98
39.81
39.64
ppm

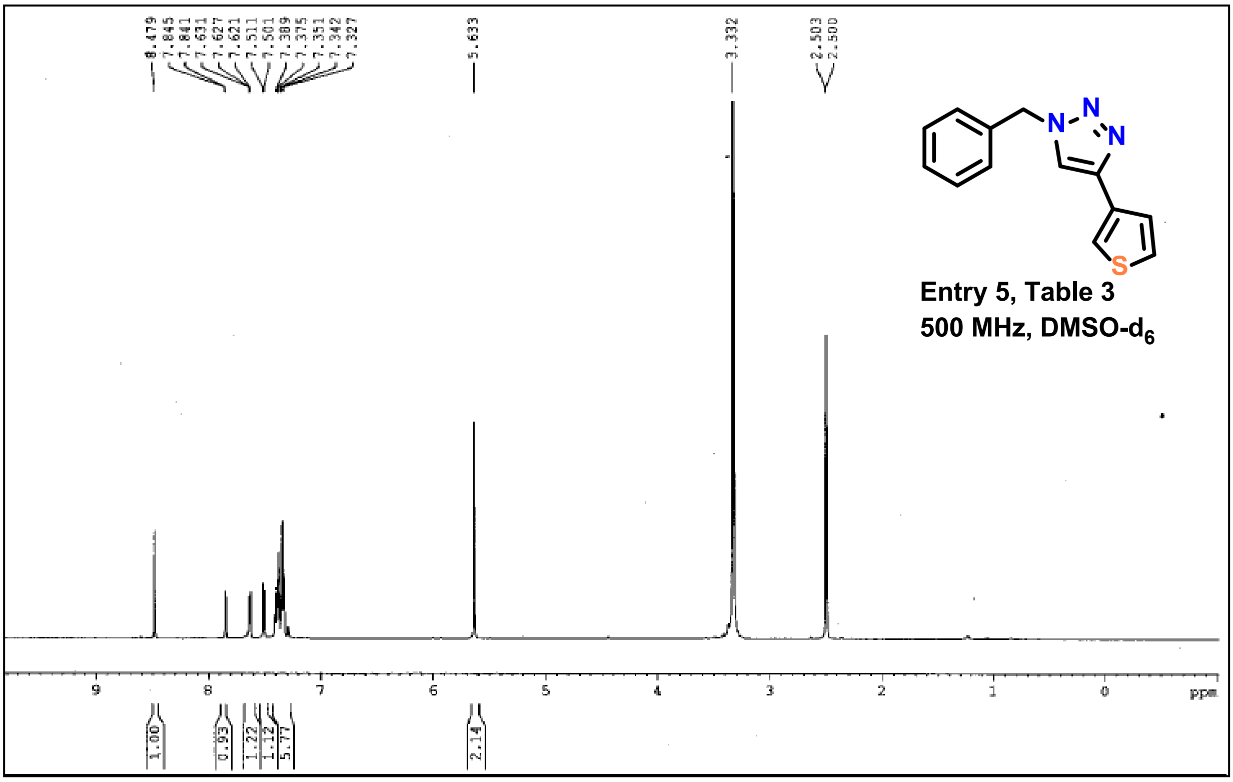
8.479
7.845
7.841
7.631
7.627
7.621
7.511
7.501
7.389
7.375
7.351
7.342
7.327
5.633
3.332
2.503
2.500
Entry 5, Table 3
500 MHz, DMSO-d6
1.00
0.93
1.22
1.12
5.77
2.14
ppm

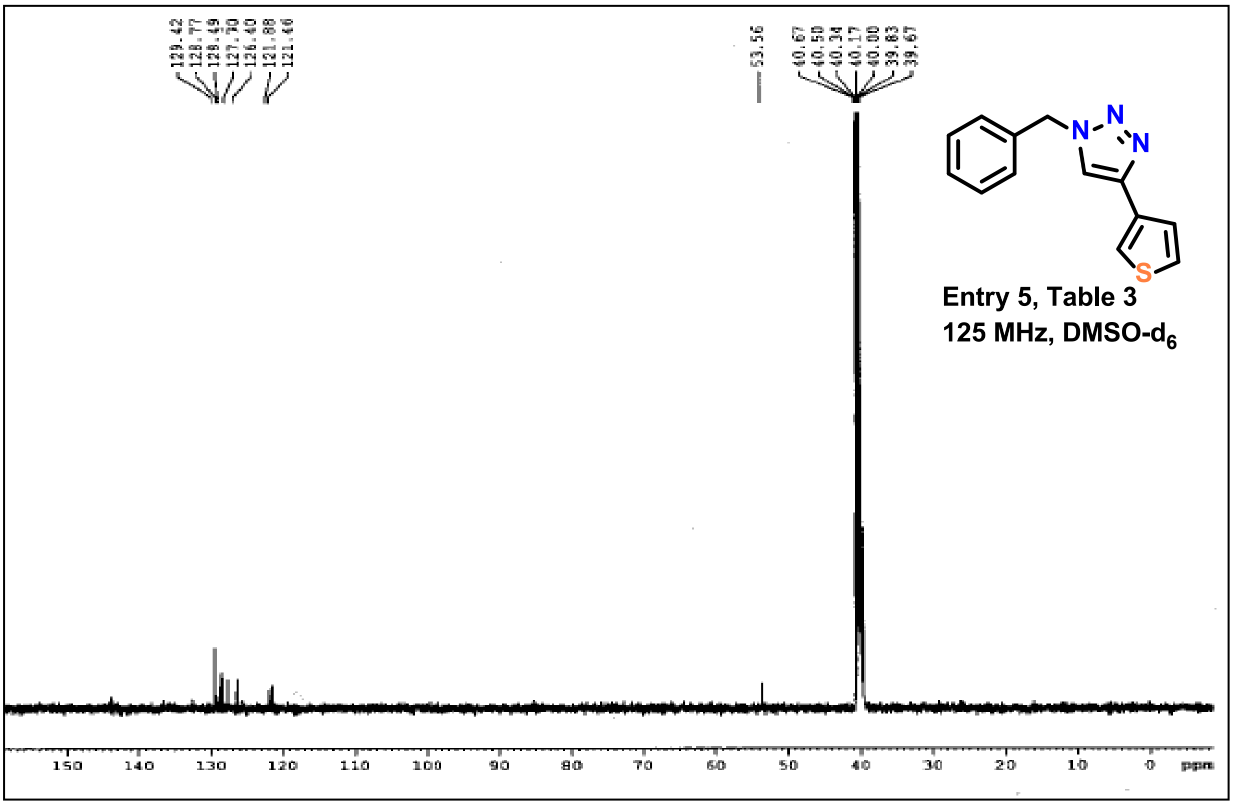
129.42
128.77
128.49
127.70
126.40
121.88
121.46
53.56
40.67
40.50
40.34
40.17
40.00
39.83
39.67
Entry 5, Table 3
125 MHz, DMSO-d6
ppm

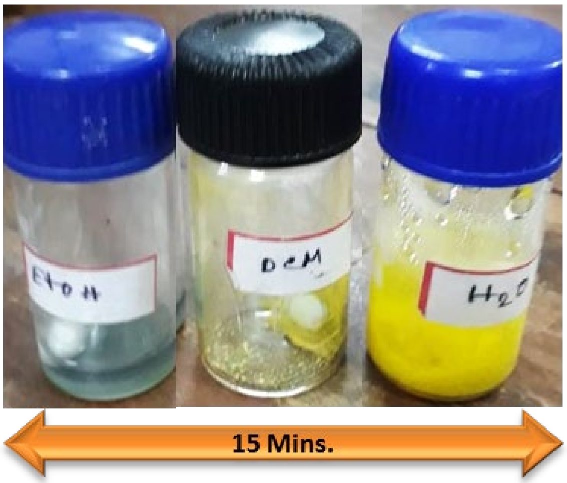

Figure S8. Solvent dependent catalytic assay of Knoevenagel Reaction after 15 min.

Figure S9. List of ^{1}H-NMR and ^{13}C-NMR spectroscopy of Knoevenagel Reactions.

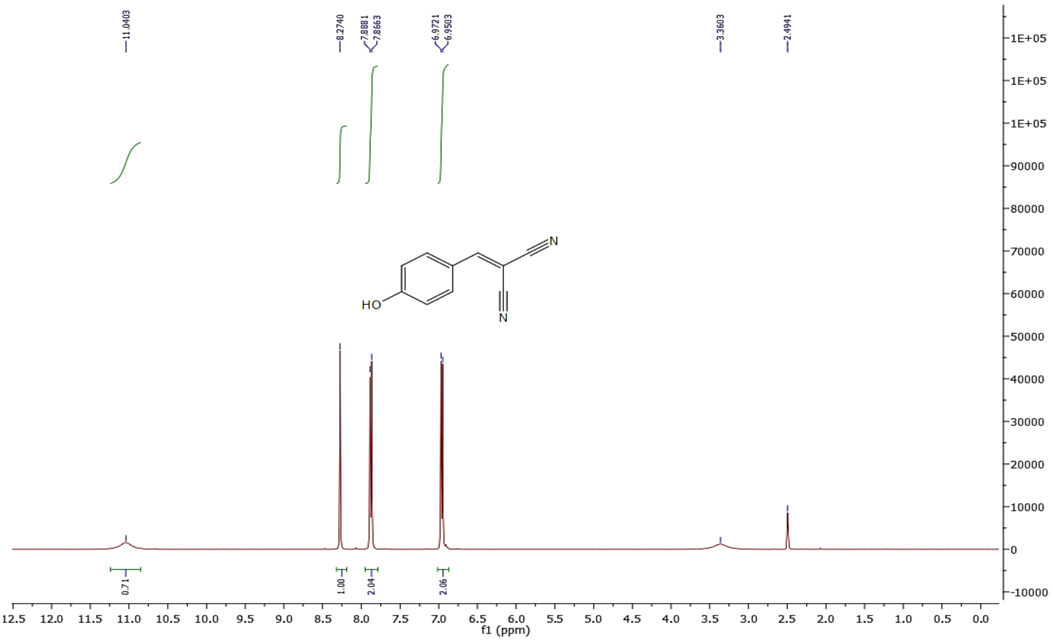

Entry 1, Table 4:

^{1}H NMR (400 MHz, DMSO-d_6) δ: 11.04 (1 H, bs), 8.27 (1 H, s), 7.88 (2 H, d, *J* 8.7), 6.96 (2 H, d, *J* 8.7).

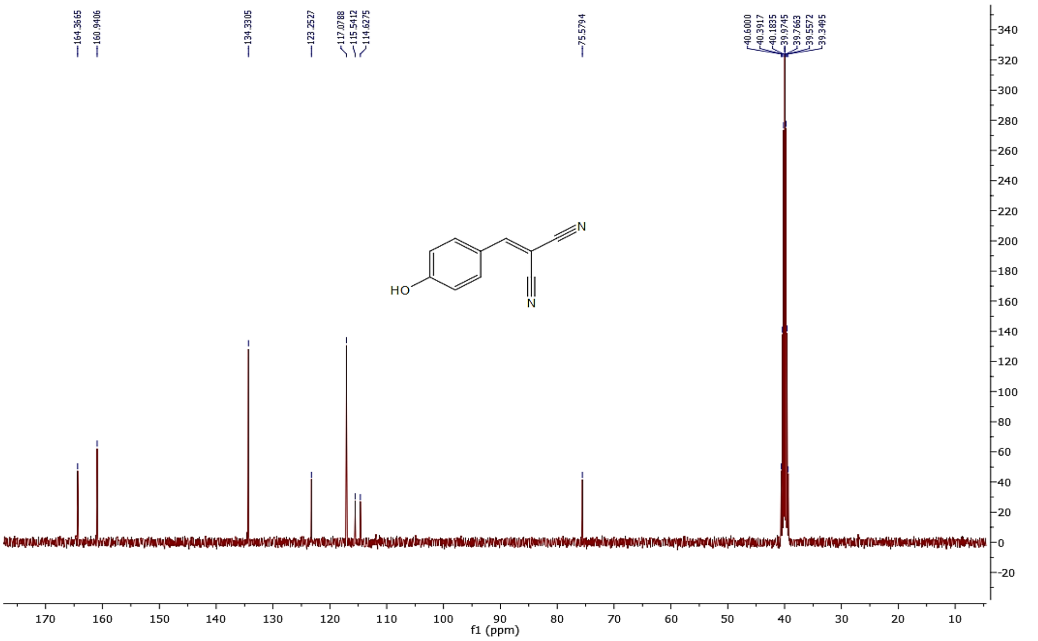

Entry 1, Table 4:

^{13}C NMR (100 MHz, DMSO-d$_6$) δ: 164.37, 160.94, 134.33, 123.25, 117.08, 115.54, 114.63, 75.58.

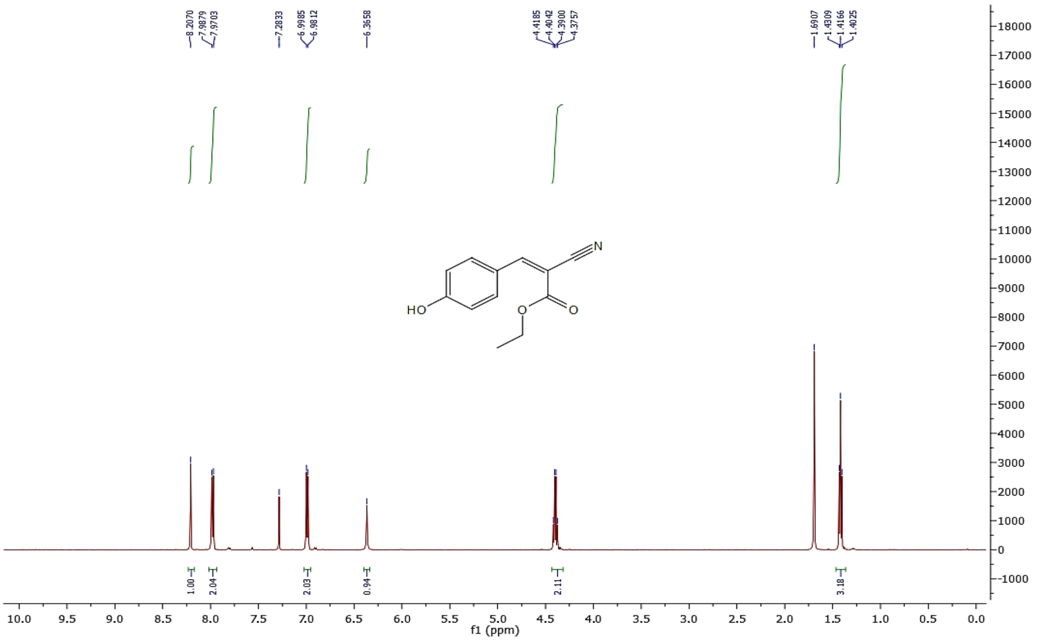

Entry 2, Table 4:

^{1}H NMR (500 MHz, CDCl$_3$) δ: 8.21 (1 H, s), 7.98 (2 H, d, *J* 8.8), 6.99 (2 H, d, *J* 8.7), 6.37 (1 H, s), 4.40 (2 H, q, *J* 7.1), 1.42 (3 H, t, *J* 7.1).

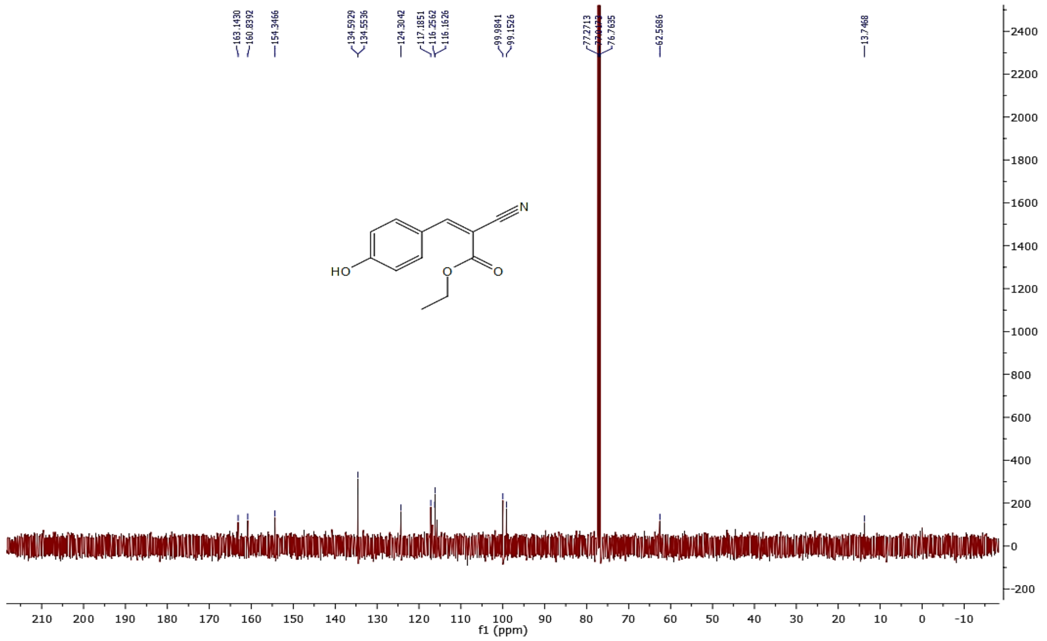

Entry 2, Table 4:

^{13}C NMR (125 MHz, $CDCl_3$) δ: 163.14, 160.84, 154.35, 134.59, 134.55, 124.30, 117.19, 116.26, 116.16, 99.98, 99.15, 62.57, 13.75.

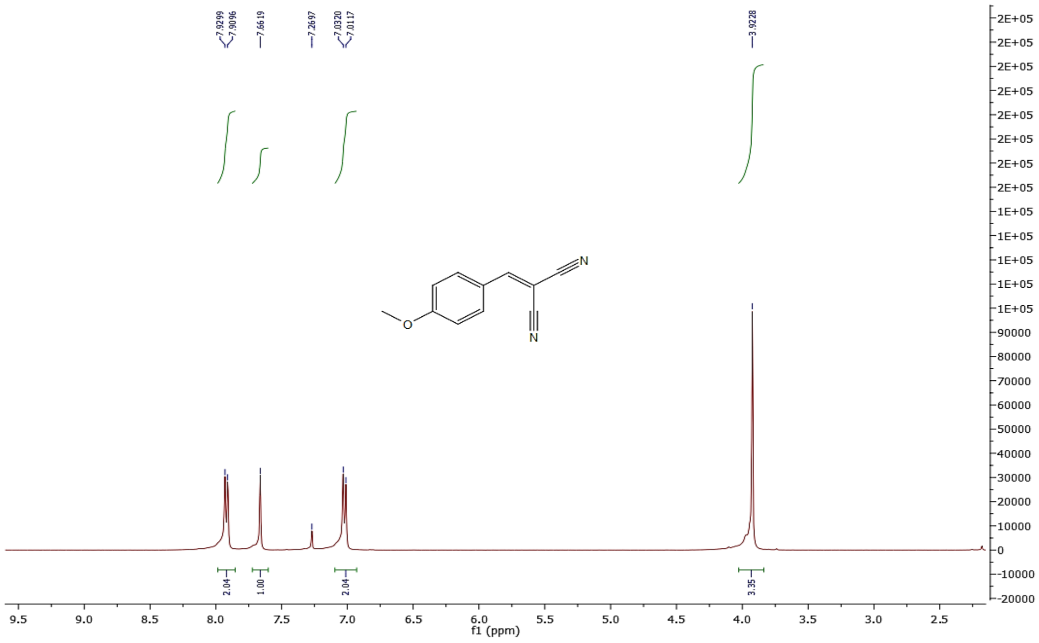

Entry 3, Table 4:

^{1}H NMR (400 MHz, $CDCl_3$) δ: 7.92 (2 H, d, *J* 8.1), 7.66 (1 H, s), 7.02 (2 H, d, *J* 8.1), 3.92 (3 H, s).

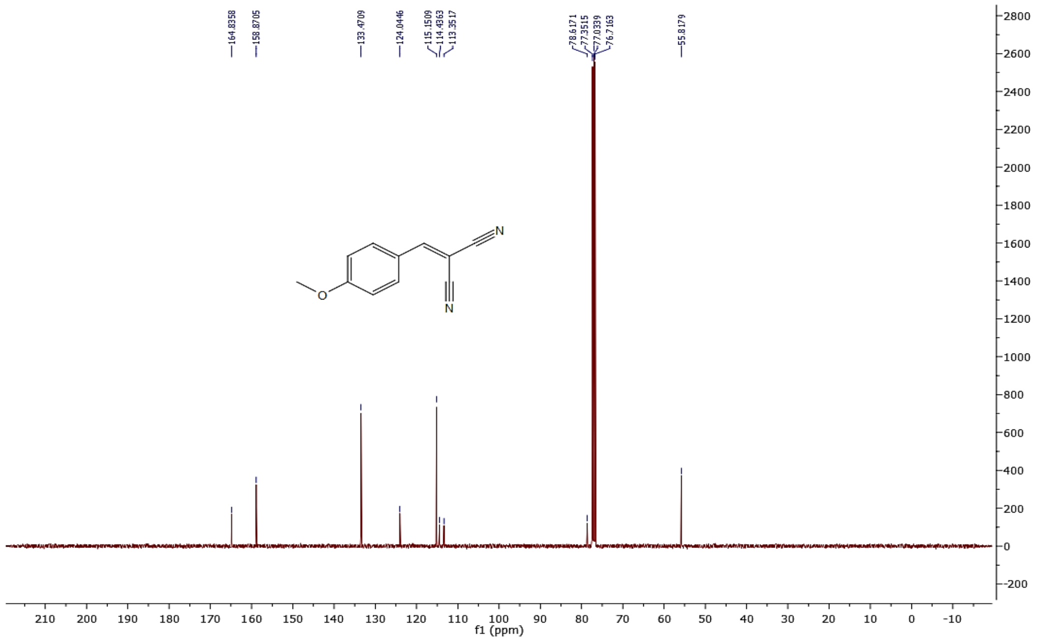

Entry 3, Table 4:
^{13}C NMR (100 MHz, $CDCl_3$) δ: 164.84, 158.87, 133.47, 124.04, 115.15, 114.44, 113.35, 78.62, 55.82.

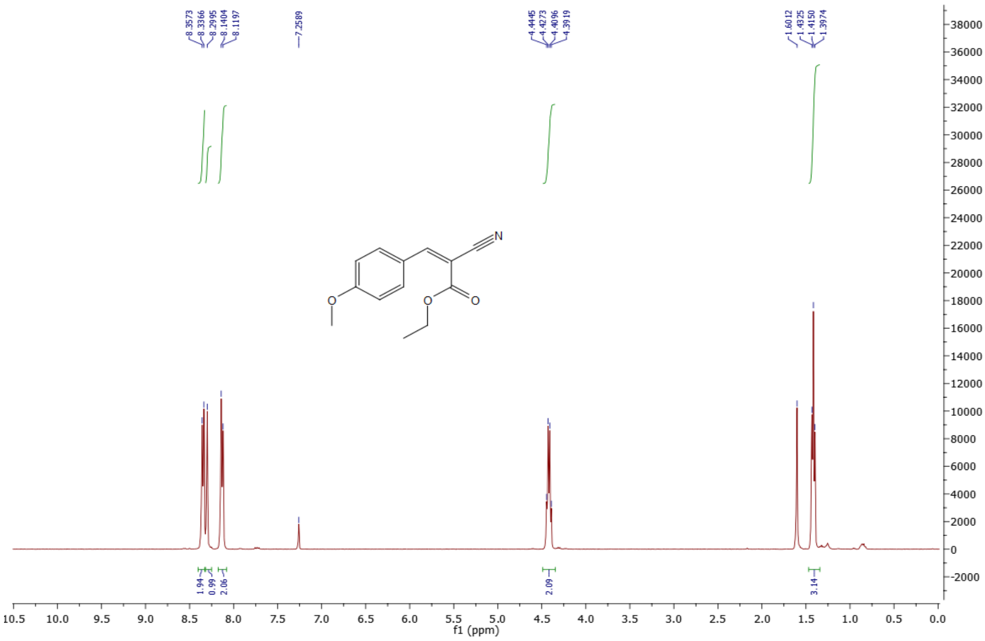

Entry 4, Table 4:
^{1}H NMR (400 MHz, $CDCl_3$) δ: 8.35 (2 H, d, *J* 8.3), 8.30 (1 H, s), 8.13 (2 H, d, *J* 8.3), 4.42 (2 H, q, *J* 7.0), 1.41 (3 H, t, *J* 7.0).

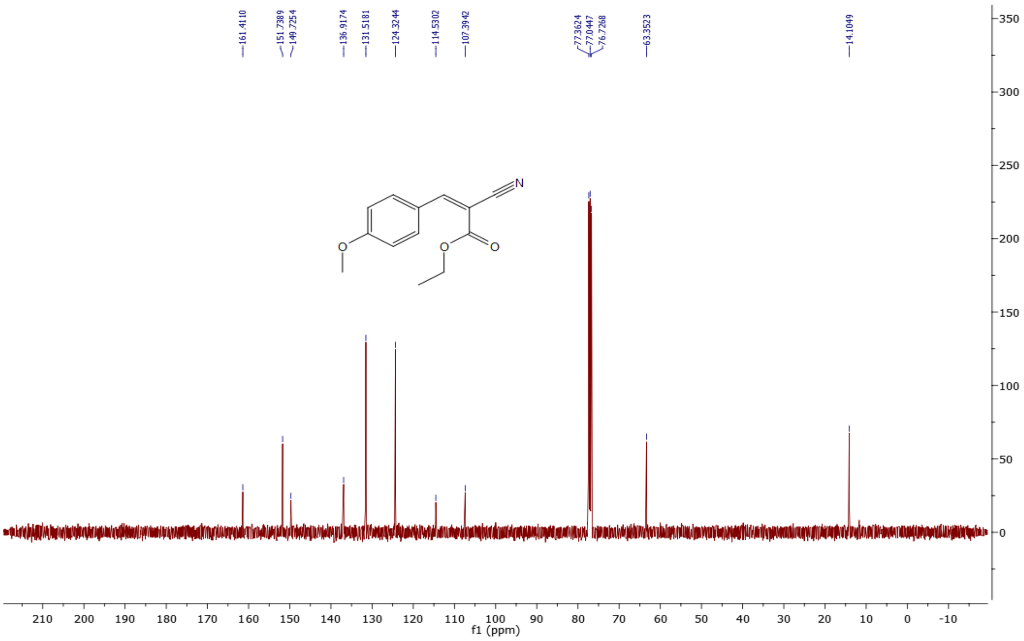

Entry 4, Table 4:

^{13}C NMR (100 MHz, $CDCl_3$) δ: 161.41, 151.74, 149.73, 136.92, 131.52, 124.32, 114.53, 107.39, 63.35, 14.10.

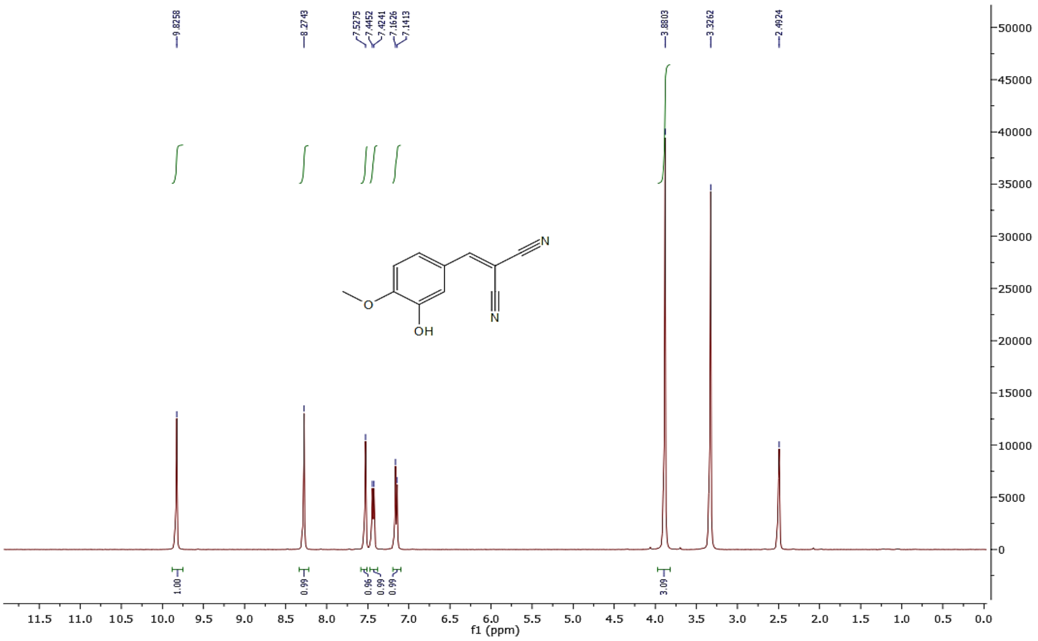

Entry 5, Table 4:

^{1}H NMR (400 MHz, DMSO d_6) δ: 9.83 (1 H, s), 8.27 (1 H, s), 7.53 (1 H, s), 7.43 (1 H, d, *J* 8.4), 7.15 (1 H, d, *J* 8.5), 3.88 (3 H, s).

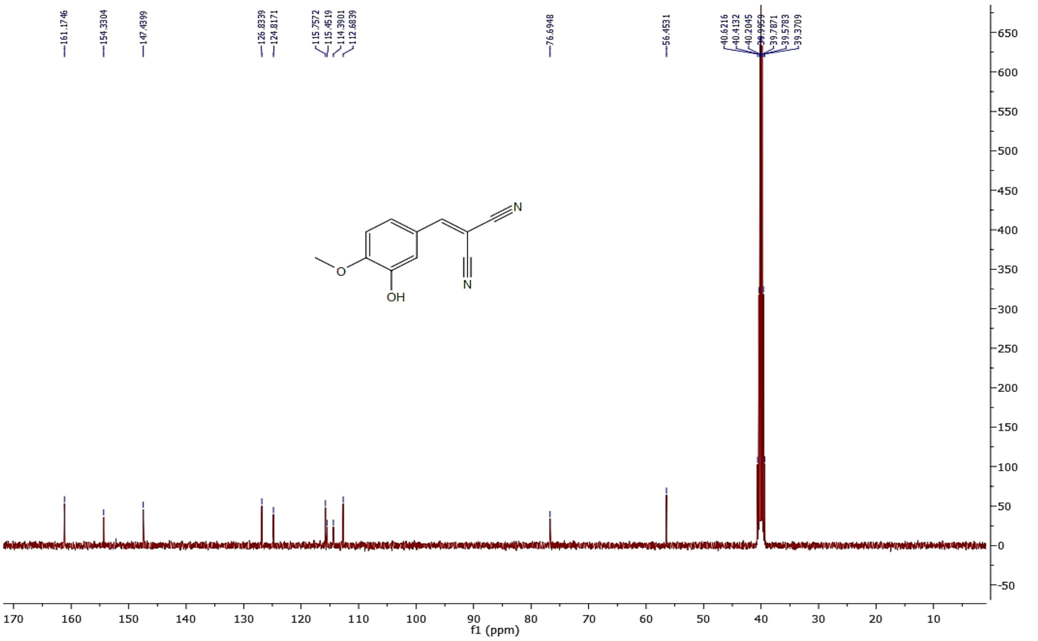

Entry 5, Table 4:

^{13}C NMR (100 MHz, DMSO d$_6$) δ: 161.17, 154.33, 147.44, 126.83, 124.82, 115.76, 115.45, 114.39, 112.68, 76.69, 56.45.

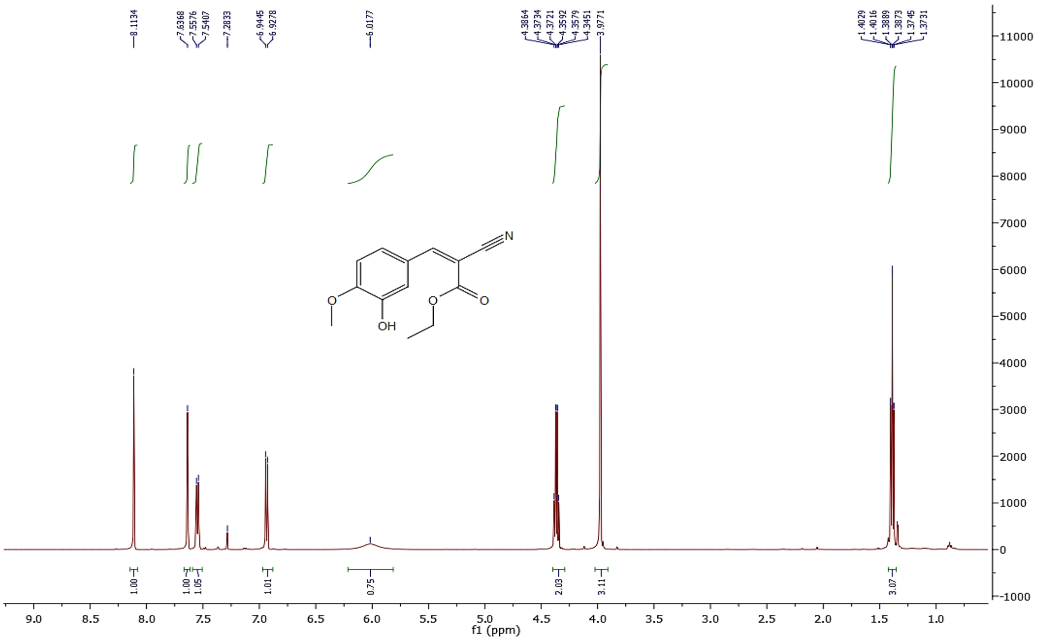

Entry 6, Table 4:

^{1}H NMR (500 MHz, $CDCl_3$) δ: 8.11 (1 H, s), 7.64 (1 H, s), 7.55 (1 H, d, *J* 8.5), 6.94 (1 H, d, *J* 8.4), 6.02 (1 H, bs), 4.39 – 4.35 (2 H, m), 3.98 (3 H, s), 1.39 (3 H, td, *J* 7.0, 0.7).

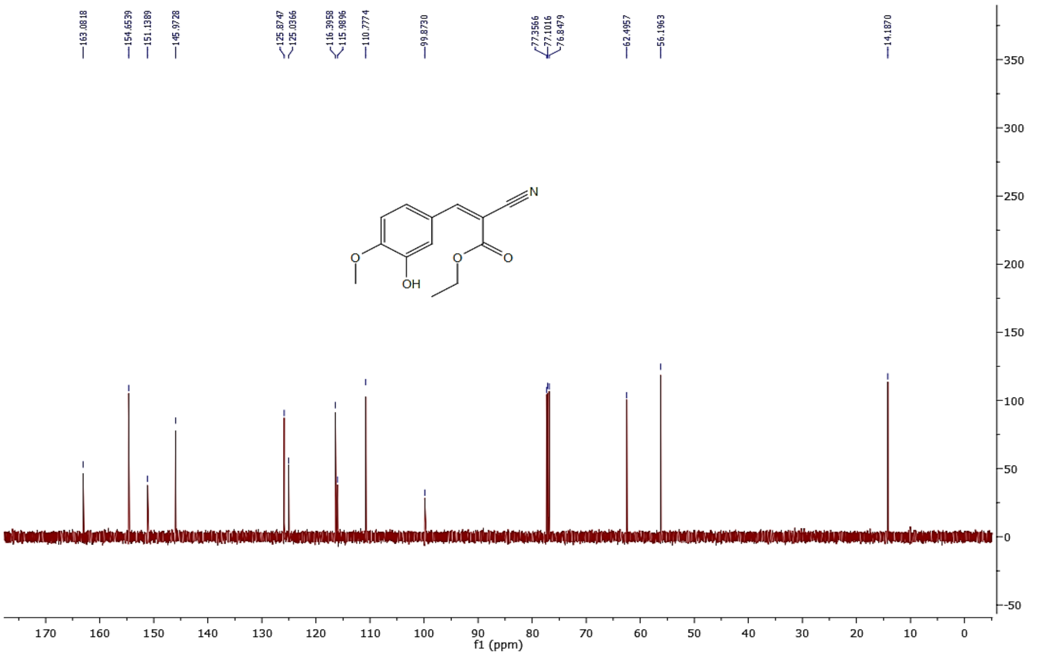

Entry 6, Table 4:

^{13}C NMR (125 MHz, $CDCl_3$) δ: 163.08, 154.65, 151.14, 145.97, 125.87, 125.04, 116.40, 115.99, 110.78, 99.87, 62.50, 56.20, 14.19.

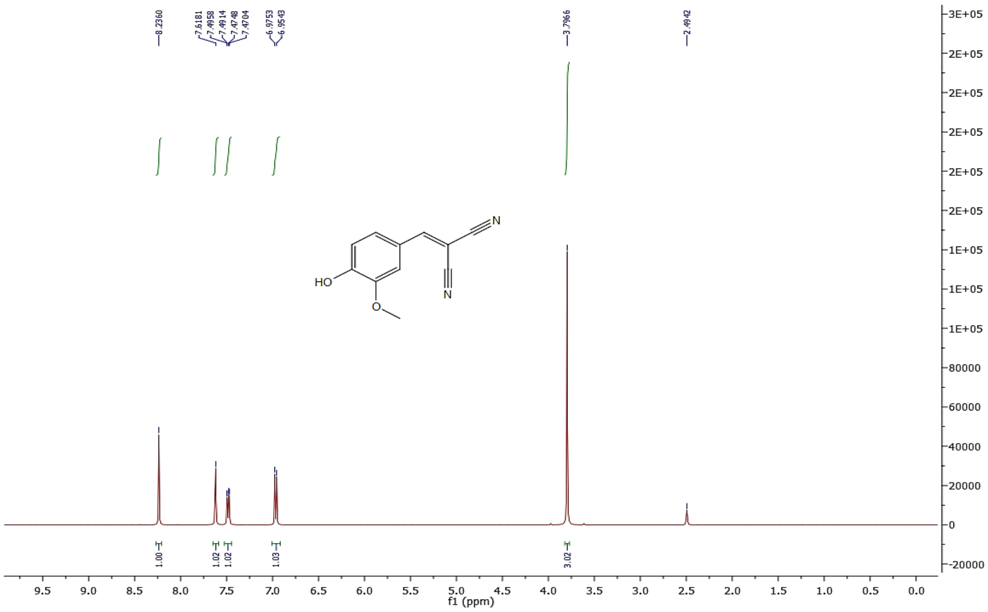

Entry 7, Table 4:

^{1}H NMR (400 MHz, DMSO-d_6) δ: 8.24 (1 H, s), 7.62 (1 H, s), 7.48 (1 H, dd, *J* 8.4, 1.7), 6.96 (1 H, d, *J* 8.4), 3.80 (3 H, s).

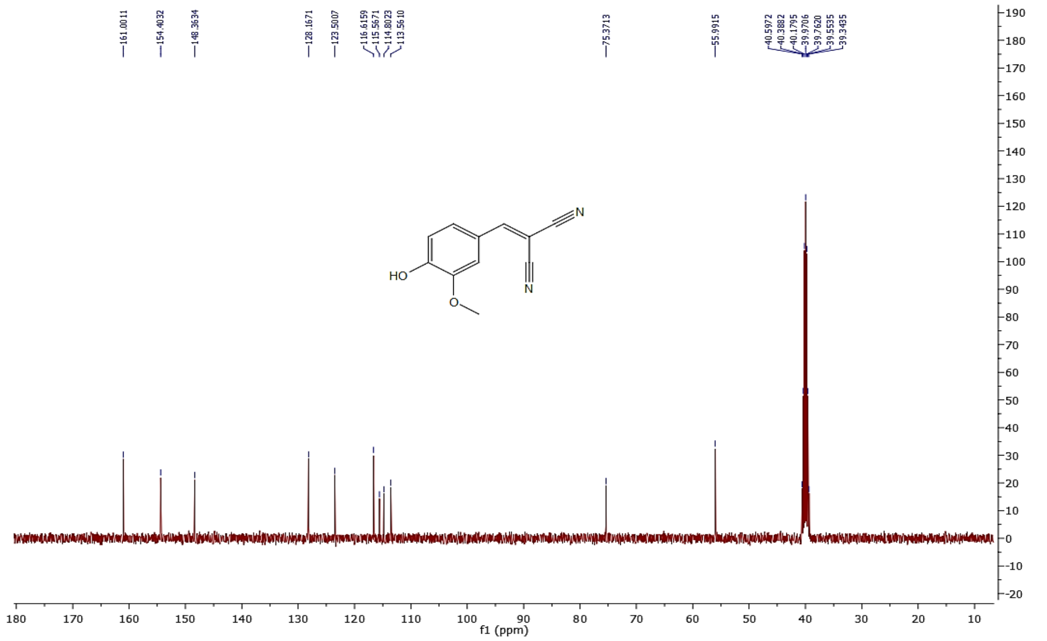

Entry 7, Table 4:

^{13}C NMR (100 MHz, DMSO-d$_6$) δ: 161.00, 154.40, 148.36, 128.17, 123.50, 116.62, 115.57, 114.80, 113.56, 75.37, 55.99.

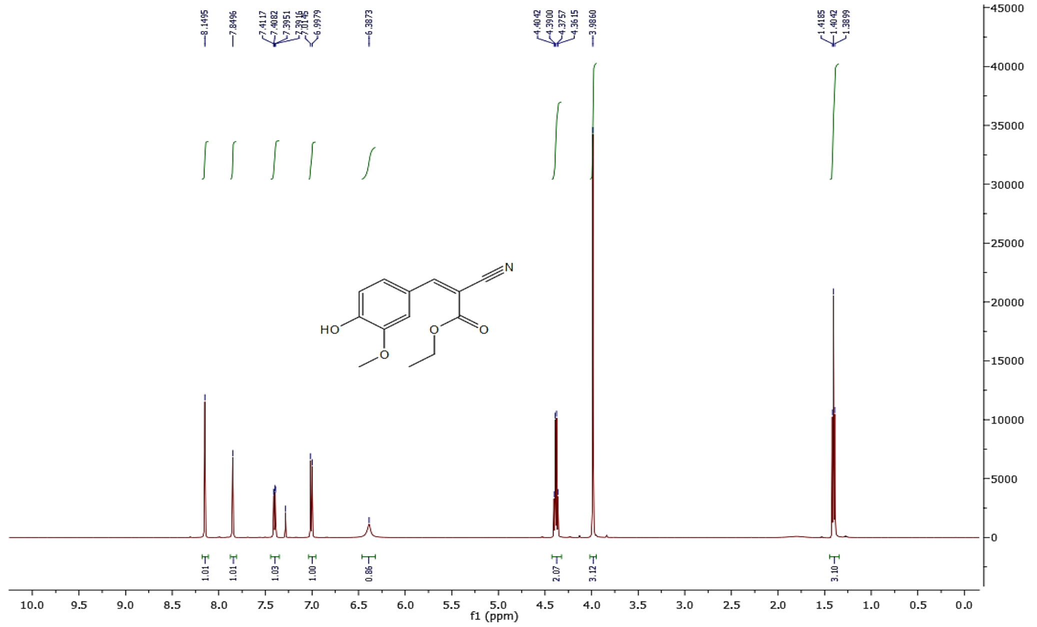

Entry 8, Table 4:

^{1}H NMR (500 MHz, $CDCl_3$) δ: 8.15 (1 H, s), 7.85 (1 H, s), 7.40 (1 H, dd, *J* 8.3, 1.8), 7.01 (1 H, d, *J* 8.3), 6.39 (1 H, bs), 4.38 (2 H, q, *J* 7.1), 3.99 (3 H, s), 1.40 (3 H, t, *J* 7.1).

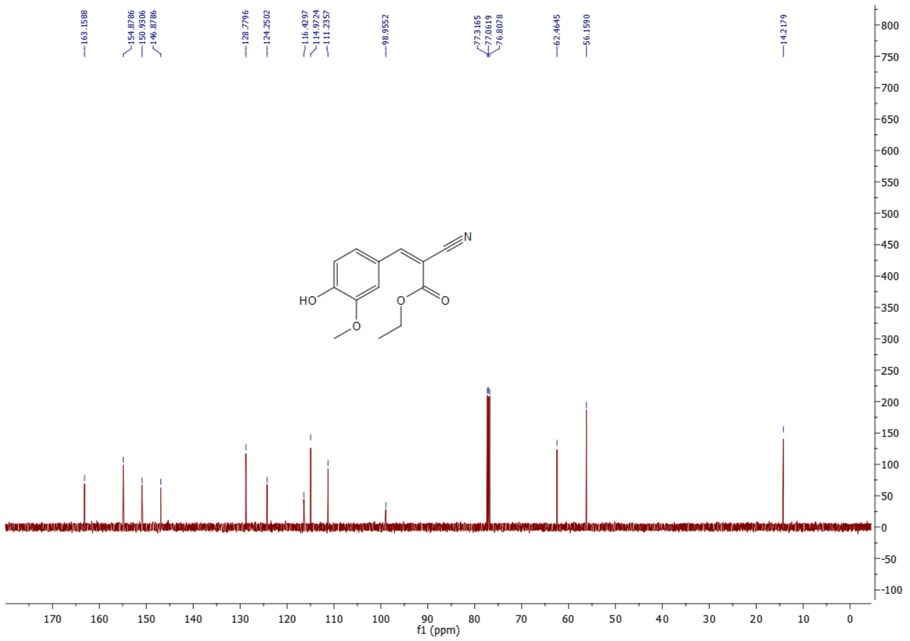

Entry 8, Table 4:

^{13}C NMR (125 MHz, $CDCl_3$) δ: 163.16, 154.88, 150.93, 146.88, 128.78, 124.25, 116.43, 114.97, 111.24, 98.96, 62.46, 56.16, 14.22.

References

1. Yang, Y.; Yao, H. F.; Xi, F. G.; Gao, E. Q. Amino-functionalized Zr(IV) metal–organic framework as bifunctional acid–base catalyst for Knoevenagel condensation. *J. Mol. Catal. A*, **2014**, *390*, 198–205.
2. Valvekens, P.; Vandichel, M.; Waroquier, M.; Van Speybroeck, V.; De Vos, D. Metal-dioxidoterephthalate MOFs of the MOF-74 type: Microporous basic catalysts with well-defined active sites. *J. Catal*. **2014**, *317*, 1–10.
3. Valvekens, P.; Jonckheere, D.; De Baerdemaeker, T.; Kubarev, A.; Vandichel, M.; Hemelsoet, K.; Waroquier, M.; Van Speybroeck, V.; Smolders, E.; Depla, D.; Roeffaers, M. B. J.; De Vos, D. Base catalytic activity of alkaline earth MOFs: a (micro)spectroscopic study of active site formation by the controlled transformation of structural anions. *Chem. Sci*. **2014**, *5*, 4517–4524.
4. Song, X.; He, Y.; Cai, Z.; Li, X.; Sun, Y.; Liu, H.; Lu, Y.; Hou, J.; Han, E. Three Amino-functionalized Alkaline Earth Metal-Organic Frameworks as Catalysts for Knoevenagel Condensation. *Chemistry Select*, **2020**, *5*, 11510– 11516.
5. Lin, X.-M.; Li, T.-T.; Chen, L.-F.; Zhang, L.; Su, C.-Y. Two ligand-functionalized Pb(II) metal–organic frameworks: structures and catalytic performances. *Dalton Trans.*, **2012**, *41*, 10422–10429.
6. Hasegawa, S.; Horike, S.; Matsuda, R.; Furukawa, S.; Mochizuki, K.; Kinoshita, Y.; Kitagawa, S. Three-Dimensional Porous Coordination Polymer Functionalized with Amide Groups Based on Tridentate Ligand: Selective Sorption and Catalysis. *J. Am. Chem. Soc.*, **2007**, *129*, 2607–2614.

7. Sharma, M. K.; Singh, P. P.; Bharadwaj, P. K. Two-dimensional rhombus grid coordination polymer showing heterogeneous catalytic activities. *J. Mol. Catal. A: Chem*. **2011**, *342–343*, 6–10.
8. Das, R. K.; Aijaz, A.; Sharma, M. K.; Lama, P.; Bharadwaj, P. K. Direct Crystallographic Observation of Catalytic Reactions inside the Pores of a Flexible Coordination Polymer. *Chem. Eur. J.*, **2012**, *18*, 6866–6872.
9. Wang, J.-S.; Jin, F.-Z.; Ma, H.-C.; Li, X.-B.; Liu, M.-Y.; Kan, J.-L.; Chen, G.-J.; Dong, Y.-B. Au@Cu(II)-MOF: Highly Efficient Bifunctional Heterogeneous Catalyst for Successive Oxidation–Condensation Reactions. *Inorg. Chem.*, **2016**, *55*, 6685–6691.
10. R. A. Agarwal and S. Mukherjee, Two-dimensional flexible Ni(II)-based porous coordination polymer showing single-crystal to single-crystal transformation, selective gas adsorption and catalytic properties. *Polyhedron*, **2016**, *105*, 228–237.
11. Joharian, M.; Morsali, A.; Tehrani, A. A.; Carlucci, L.; Proserpio, D.M. Water-stable fluorinated metal–organic frameworks (F-MOFs) with hydrophobic properties as efficient and highly active heterogeneous catalysts in aqueous solution. *Green Chem.*, **2018**, *20*, 5336–5345.
12. Schejn, A.; Mazet, T.; Falk, V.; Balan, L.; Aranda, L.; Medjahdi, G.; Schneider, R. Fe_3O_4@ZIF-8: magnetically recoverable catalysts by loading Fe_3O_4 nanoparticles inside a zinc imidazolate framework *Dalton Trans.*, **2015**, *44*, 10136–10140.
